# Reversible quantitative guest sensing *via* spin crossover of an iron(ii) triazole†
†Electronic supplementary information (ESI) available: Synthetic details, NMR spectra, instrumentation, plus additional structural, magnetic and TGA information and diagrams. CCDC 1434103 and 1434104. For ESI and crystallographic data in CIF or other electronic format see DOI: 10.1039/c5sc04583e


**DOI:** 10.1039/c5sc04583e

**Published:** 2016-02-09

**Authors:** Reece G. Miller, Sally Brooker

**Affiliations:** a Department of Chemistry and MacDiarmid Institute for Advanced Materials and Nanotechnology , University of Otago , PO Box 56 , Dunedin , 9054 New Zealand . Email: sbrooker@chemistry.otago.ac.nz ; Fax: +64 3 4797906 ; Tel: +64 3 4797919

## Abstract

A new phenyl-triazole-pyrazine ligand, 4-*p*-tolyl-3-(phenyl)-5-(2-pyrazinyl)-1,2,4-triazole (**tolpzph**), was prepared in order to enforce pyrazine coordination of the iron(ii) centre in the resulting complex, [Fe^II^(**tolpzph**)_2_(NCS)_2_]·THF (**1**·THF). Structure determinations carried out on this discrete mononuclear complex, **1**·THF, at 273 K (mostly high spin) and 100 K (mostly low spin) demonstrate this was successful, and that spin crossover (SCO) occurred on cooling. Subsequent magnetic measurements on **1**·THF revealed that it shows highly sensitive and reversible solvent-dependent SCO, with *T*_1/2_(**1**·THF) = 255 K *vs. T*_1/2_(**1**) = 212 K (with SCO of **1** more abrupt and occurring with a 4 K hysteresis loop), a drop of 43 K due to THF loss. This is reversible over at least 10 cycles of re-solvating with THF followed by re-drying, so **1** ↔ **1**·THF can be considered an ‘on–off’ THF sensor, monitored by the *T*_1/2_ reversibly shifting (by 43 K). Furthermore, *quantitative sensing* of the fractional amount of THF present in **1**·*n*THF, 0 ≤ *n* ≤ 1, is demonstrated. Monitoring the *T*_1/2_ and using TGA to quantify *n*(THF) revealed a linear dependence (25 data points; Pearson *r*^2^ = 0.93): *T*_1/2_ = 41.1*n*(THF) + 219. Finally, **1** is also shown to take up CHCl_3_ [*T*_1/2_(**1**·CHCl_3_) = 248 K], with a logarithmic *T*_1/2_ dependence on the fractional amount of CHCl_3_ present (10 data points; Pearson *r*^2^ = 0.98): *T*_1/2_ = 27.0 log_10_[*n*(CHCl_3_)] + 243. This study is a proof of principle that a (multi-use) *quantitative sensor* material based on spin crossover is feasible.

## Abstract 2

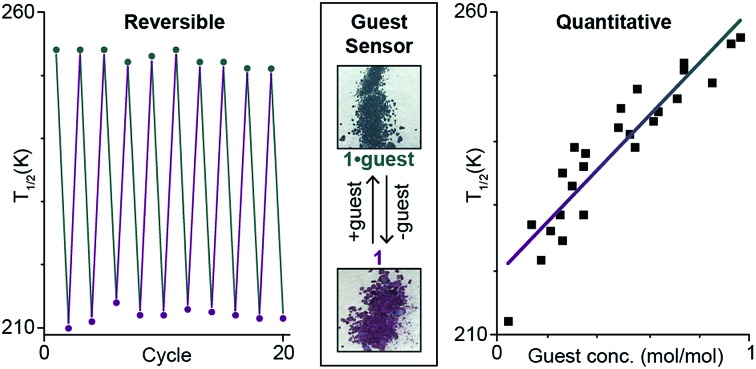
Discrete mononuclear [Fe^II^(**tolpzph**)_2_(NCS)_2_]·THF (**1**·THF), shows highly sensitive, robust and reversible solvent-dependent spin crossover, enabling it to act as a *quantitative* small molecule sensor.

## Introduction

Spin crossover (SCO) can be observed in d^4^–d^7^ transition metal complexes when the ligand field around the metal centre is perfectly tuned, such that application of an external stimulus causes the complex to switch between the low spin (LS) and high spin (HS) states.^1^–^6^ SCO-active materials have primarily been of interest due to the potential to store information when very slow relaxation (HS ↔ LS) allows a metastable state (within a hysteresis loop) to be observed for a finite lifetime.^4^–^8^

Due to the sensitivity of SCO-active materials to their environment, sensor applications are also of considerable interest, as exemplified by a wide range of recent high profile reports.^9^–^22^ These examples have focussed on the use of SCO as a *qualitative* on/off-sensor,^9^,^11^,^13^,^16^,^19^–^24^ in some cases showing remarkable tuning of the spin crossover *T*_1/2_ (which can be monitored by many techniques besides magnetic, such as IR and UV-Vis spectroscopy) by varying the type of guest molecule within the crystal lattice.^5^,^9^,^11^,^13^,^19^–^22^,^25^ Metal–organic frameworks (MOFs) with permanent porosity have been prominent in such studies.^9^,^11^,^13^,^22^,^26^–^28^ Similarly, stepwise tuning of *T*_1/2_ by post-synthetic modification of a porous MOF has been reported.^29^ Whilst on/off sensing is inherently useful, for many applications a *quantitative* sensor would have significant advantages. A couple of solution iron(ii) SCO-systems have shown some promise in this regard, including proof of concept studies showing sensing of anions in non-protic solvents^30^–^32^ or of sensing temperature in aqueous solution [for PARACEST (paramagnetic chemical exchange saturation transfer) MRI thermometers].^33^

Reported herein is the synthesis, structure and magnetic properties of [Fe^II^(**tolpzph**)_2_(NCS)_2_]·THF, **1**·THF, which can be *reversibly desolvated* to give **1**. Both **1**·THF and **1** are shown to be SCO-active but the *T*_1/2_ values differ by 43 K. To the best of our knowledge we report herein the first demonstration of *reversible quantitative guest sensing by an SCO-active material*.

**Scheme 1:**
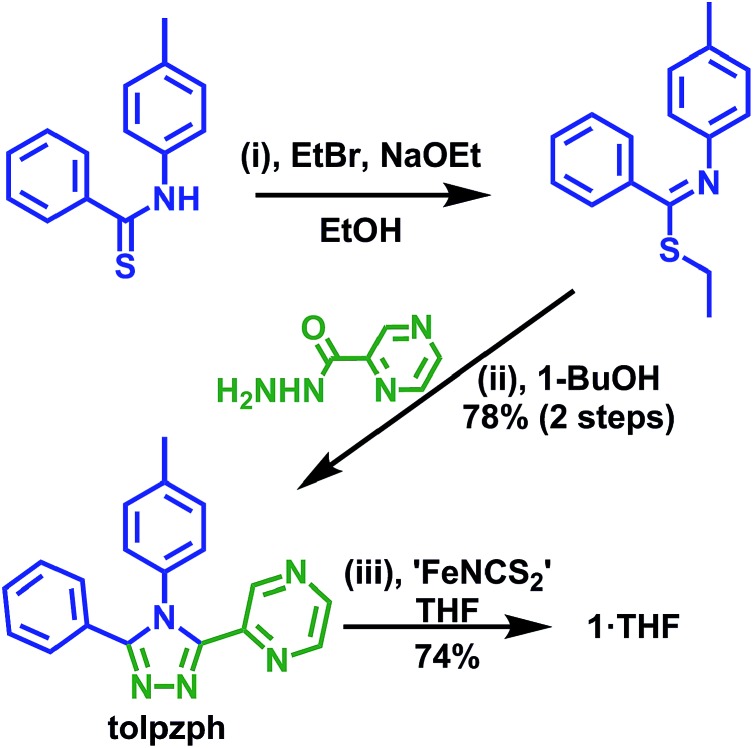
Synthesis of the novel ligand **tolpzph** and the iron(ii) isothiocyanato complex of it [Fe^II^(**tolpzph**)_2_(NCS)_2_]·THF (**1**·THF).

Our previous work with the ligand 4-*p*-tolyl-3-(2-pyrazinyl)-5-(2-pyridyl)-1,2,4-triazole (**tolpzpy**, Fig. S7†) resulted in an unexpected preference of the iron(ii) centre for the better σ-donor but poorer π-acceptor^34^,^35^ pyridine donor over the pyrazine donor.^36^ This led us to develop an analogue, the pyrazine-phenyl ligand **tolpzph** (Scheme 1), in order to facilitate coordination of the iron(ii) centre to the pyrazine moiety by removing the competition provided by the pyridine moiety, replacing it with a phenyl ring.

## Results and discussion

### Synthesis

The new ligand **tolpzph** is prepared in excellent yield (78% over two steps) from the known precursor *N*-(*p*-tolyl)-benzenethioamide using methods adapted from our previously reported syntheses (Scheme 1).^36^,^37^

The complex **1**·THF is prepared in good yield (74%), under Schlenk conditions, by the *in situ* preparation of ‘Fe^II^(NCS)_2_’ in dry THF followed by the addition of a dry THF solution of **tolpzph**. Interestingly, unlike the previously reported complex of the pyrazine-pyridine ligand, [Fe^II^(**tolpzpy**)_2_(NCS)_2_],^36^ the synthesis of **1**·THF is highly moisture sensitive. The complex also readily decomposes in solution in the presence of coordinating solvents such as MeOH or MeCN. These observations are consistent with the reduced σ-donor character of pyrazine relative to pyridine making the Fe–N bond more labile, at least in solution. In contrast to these solution state findings, **1**·THF is stable in air in the solid state.

### Structure determinations at 273 and 100 K

**Fig. 1:**
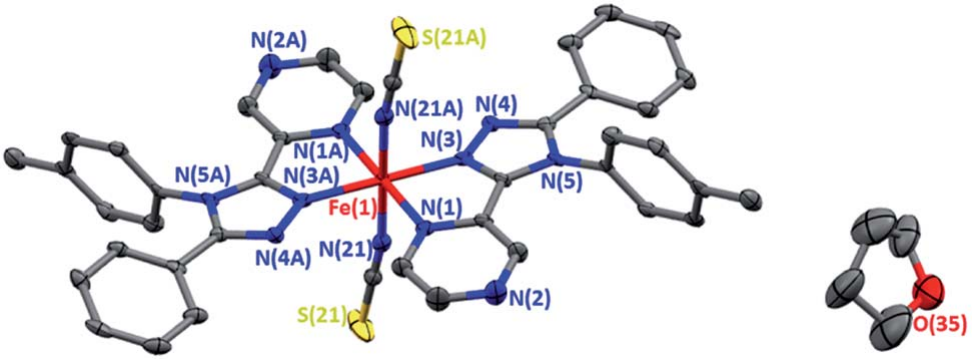
Solid state structure of [Fe^II^(**tolpzph**)_2_(NCS)_2_]·THF (**1**·THF) at 100 K. Only one of the disordered 1/4 occupancy THF molecules shown; H atoms omitted for clarity; thermal ellipsoids drawn at 70% probability.

Forest green, rod shaped, single crystals of **1**·THF were grown by vapour diffusion of dry, degassed diethyl ether into a THF solution of the reaction mixture under an inert atmosphere. The complex crystallises in the orthorhombic space group *Pbca*, with half of the complex in the asymmetric unit, and the other half generated by a centre of inversion at the iron(ii) centre (Fig. 1).

The Fe–N bond lengths (average 2.08 Å) and the high value of the octahedral distortion parameter (*Σ* = 63.6°) observed for **1**·THF at 273 K (Table 1) are consistent with HS iron(ii). On decreasing the temperature to 100 K the Fe–N bond lengths (average 1.97 Å) and the decreased value of the octahedral distortion parameter (*Σ* = 54.9°) are consistent with the iron(ii) centre being in the LS state (Table 1). The large decrease (8%) in the unit cell volume between 350 K and 100 K (Table 1, Fig. S17†) provides additional confirmation of the change in spin state.

**Table 1:** Selected structural data for **1**·THF at 100 K and 273 K

	100 K	273 K
Cell volume [Å^3^]	4184.24 (16)	4377.1 (2)
Fe–N_triazole_ [Å]	1.971 (3)	2.070 (2)
Fe–N_azine_ [Å]	1.999 (3)	2.125 (2)
Fe–NCS [Å]	1.951 (3)	2.033 (3)
Average Fe–N [Å]	1.97	2.08
N_triazole_–Fe–N_azine_ [°]^*a*^	80.60 (9)	77.25 (8)
N_triazole_–Fe–NCS [°]	92.35 (9)	92.11 (9)
N_azine_–Fe–NCS [°]	91.97 (9)	91.03 (9)
*Σ* [°]	54.9	63.6

^*a*^Within a ligand strand.

**Fig. 2:**
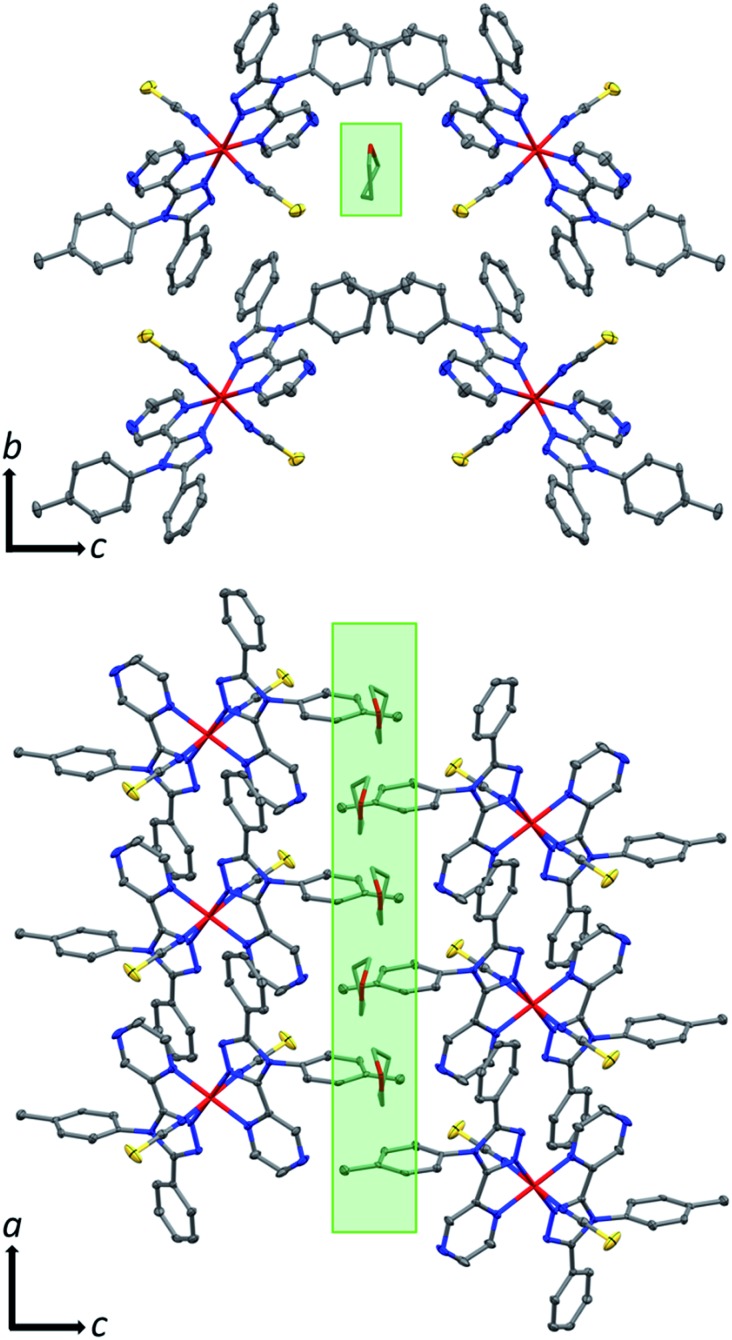
Crystal packing in **1**·THF as viewed down the crystallographic (top) *a*-axis (just 1 of the 4 overlapping (each on average 1/4 occupancy) disordered THF positions is shown for clarity) and (bottom) *b*-axis (just 2 of the 4 overlapping disordered THF positions are shown for clarity). Both views show the pore-like ‘pseudo-channels’ along the *a*-axis that contain the THF of solvation (indicated by green highlight) which can be *reversibly* removed (also see Fig. S4 and S5†). Hydrogen atoms omitted for clarity; thermal ellipsoids drawn at 70% probability. THF molecules drawn as capped sticks for clarity.

The THF molecule of solvation is disordered over two unique, overlapping, quarter occupancy sites in the asymmetric unit (Fig. S5†). This gives a total of four, one quarter occupancy, positions per iron(ii) centre. By symmetry, these form a continuous line down a ‘pseudo-channel’ along the crystallographic *a*-axis (Fig. 2, S4 and S5†).

### Magnetic characterisation

**Fig. 3:**
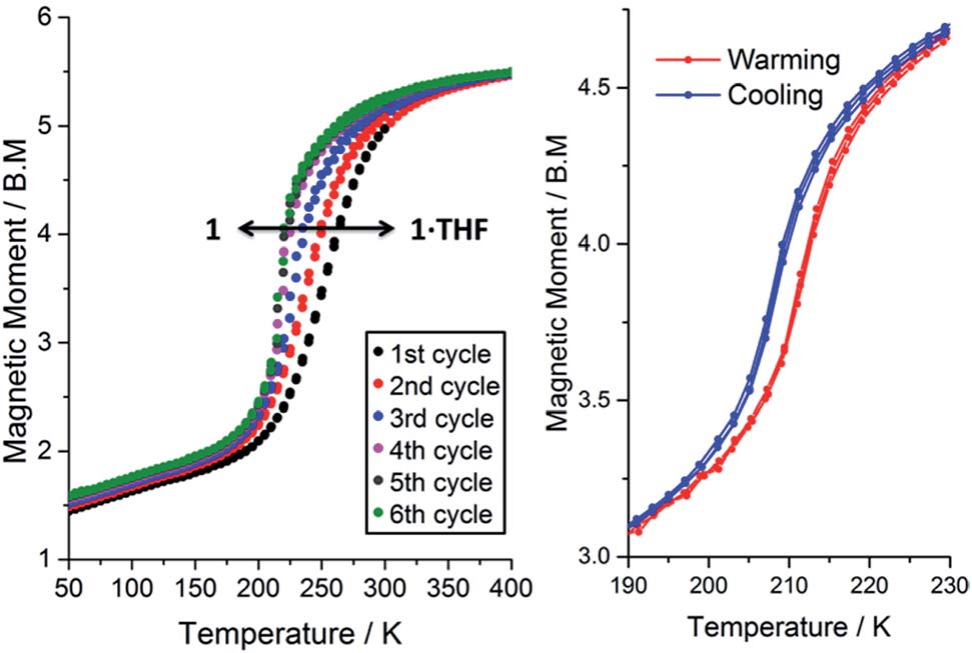
Left: Magnetic moment of **1**·*n*(THF) (where 0 ≤ *n* ≤ 1) as a function of temperature for 300 → 50 K and then repeated cycles of 50 ↔ 400 K showing the solvent dependence of SCO in this compound. Note: TGA shows that *n* = 1 for the first cycle and *n* = 0 for the 5^th^ cycle. Right: A zoom in over the temperature range of the SCO event after thorough drying (403 K, N_2(g)_, 2 hours) so that *n* = 0, shows a reproducible 4 K hysteresis (three cycles shown). All data collected in settle mode (data point taken after constant *T* to within the lesser of ±0.5% and ±0.5 K for 1 minute, see page S7†); MW and sample mass were revised for each cycle based on the relationship derived in eqn (1) (see later), but please note that using MW (**1**·THF) for all calculations makes very little difference (ESI, Fig. S28†).

The magnetic susceptibility of **1**·THF was initially studied from 300 to 50 K. At 300 K the *μ*_eff_ is 4.98 B.M., consistent with the complex being almost completely HS. On cooling, the *μ*_eff_ drops, revealing an abrupt spin transition with a *T*_1/2_ of 250 K (Fig. 3, black data points; Fig. S22†). Consistent with the crystal structure determined at 100 K (Table 1), the *μ*_eff_ of 1.63 B.M at 100 K is indicative of the complex being mostly LS.

Notably the *T*_1/2_ observed for the pyrazine-coordinated iron(ii) centre in **1**·THF is over 100 K higher than the *T*_1/2_ of 145 K observed for the related solvent-free SCO-active polymorph of [Fe^II^(**tolpzpy**)_2_(NCS)_2_] in which it is unclear whether the iron(ii) is coordinated to pyrazine or to pyridine.^36^ If the latter is pyridine bound then this is consistent with pyrazine providing a stronger ligand field than pyridine (stabilising the LS state, hence increasing the *T*_1/2_), as expected, but if both are pyrazine bound then this difference in *T*_1/2_ may well be due to the differences in crystal packing and/or solvent content.

**Fig. 4:**
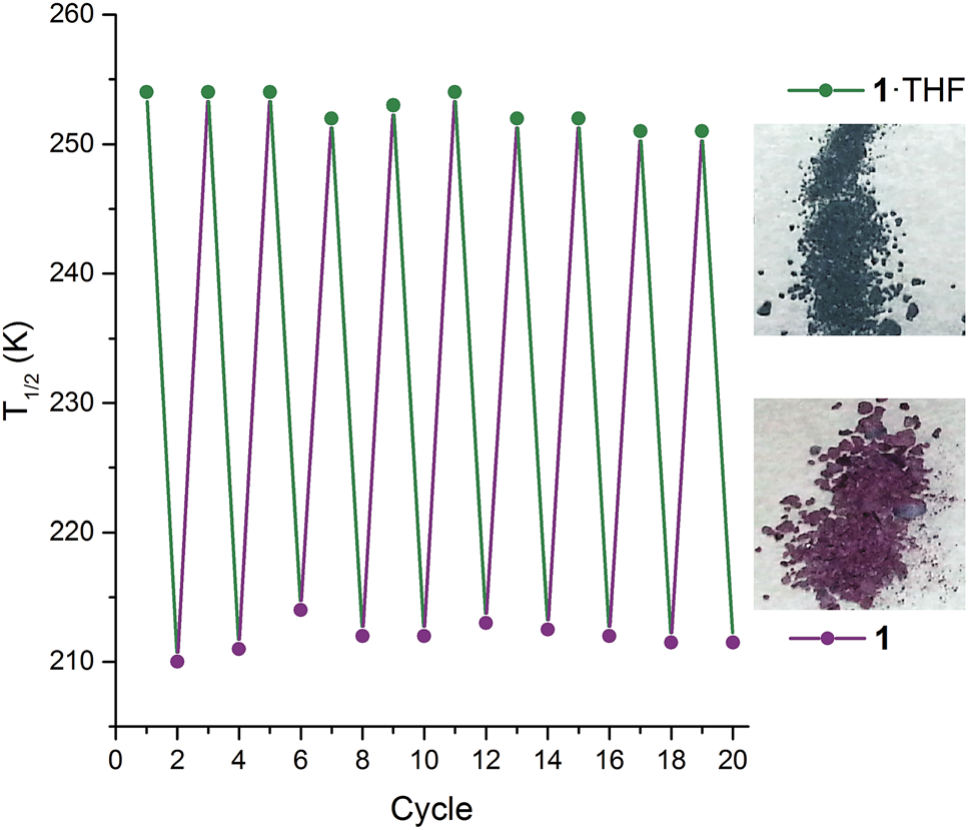
Left: The *T*_1/2_ as monitored over 10 consecutive cycles‡ of interconversions **1**·THF (green points) ↔ **1** (purple points), whereby **1**·THF is heated at 400 K for two hours to give **1** then exposed to THF vapour for 2 hours to regenerate **1**·THF (lines are simply guides to the eyes). Note: appropriate molecular weight (either **1**·THF or **1**), and revised sample mass, used for each cycle. Right: A distinct change in colour is observed between **1**·THF (forest green) and **1** (violet).

Interestingly, on repeated variable temperature cycles in the range 50–400 K, in settle mode, a small decrease in the *T*_1/2_ was observed with each cycle (Fig. 3). This was coupled with an increasingly abrupt SCO event, which by the 6^th^ cycle also shows a small (4 K) but reproducible thermal hysteresis. The changes are confirmed to be due to the loss of some THF of solvation with each cycle to high temperature by thermogravimetric analysis (TGA) after each cycle (Fig. S17†). Overall the *T*_1/2_ decreases 43 K, from *T*_1/2_(**1**·THF) = 255 K to *T*_1/2_(**1**) = 212 K. A significant solvochromic effect is also observed with a change from forest green (**1**·THF) to violet (**1**) on drying (Fig. 4, right).

The observed shift in *T*_1/2_ as the guest concentration is decreased is likely to be due to internal lattice pressure effects^6^,^13^,^38^ whereby the larger HS state becomes more favourable when the *a*-axis channels are less occupied. The observed shifting of *T*_1/2_ is *reversible* on exposure to THF vapour, for as little as 2 hours, restoring the original magnetic response *vs.* temperature. Heating the sample for 2 hours at 400 K, either under a weak vacuum (4 mbar), or under a N_2(g)_ flow, regenerates **1** and the associated magnetic response. This cycling, between **1**·THF and **1**, is reproducible over at least 10 cycles with minimal fatigue (Fig. 4, left). In summary, this **1**·THF ↔ **1** spin crossover system is a robust ‘on–off’, or *qualitative, sensor* for THF.

**Fig. 5:**
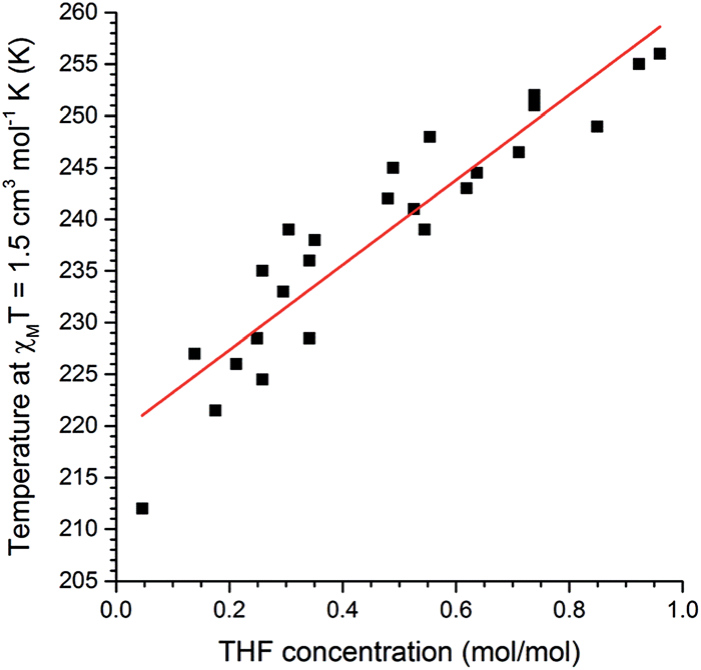
A plot of mole fraction of THF, *n*(THF) as mol(THF)/mol(Fe), as a function of the *T*_1/2_ (determined as the temperature at which *χ*_M_*T* = 1.5 cm^3^ mol^–1^ K). Pearson *r*^2^ = 0.93 for the linear fit of the 25 data points. See page S7 (and Fig. S27†) for a full description of the collection of these data.

Next, we probed whether the magnetic response could be used to determine the solvent content in **1**·*n*(THF) where this was fractional rather than simply *n* = 0 or 1, in other words whether this system can act as a *quantitative sensor* for THF. A comparison of the magnetic and thermogravimetric data reveals that the *T*_1/2_ has an approximately linear dependence on the concentration of THF guest (Fig. 5).

The relationship between *T*_1/2_ and *n*(THF) is described by the equation:

1 *T*_1/2_ = 41.1*n*(THF) + 219

with a standard error in the slope of 3.4*n*(THF), which is significantly different from 0 (*p* < 0.001). Hence this **1**·THF ↔ **1** SCO system is a *quantitative sensor* for THF.

**Fig. 6:**
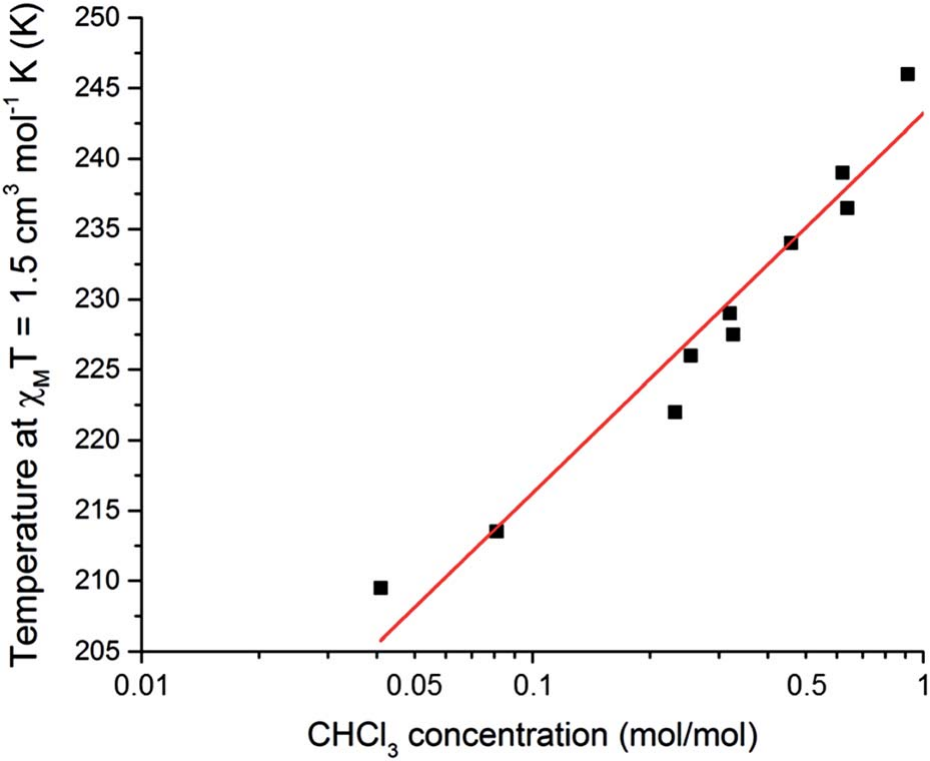
A plot of mole fraction of CHCl_3_, *n*(CHCl_3_) as mol(CHCl_3_)/mol(Fe), on a log_10_ scale as a function of the *T*_1/2_ (determined as the temperature at which *χ*_M_*T* = 1.5 cm^3^ mol^–1^ K). Pearson *r*^2^ = 0.98 for the linear fit of the 10 data points. See page S7 (and Fig. S27†) for a full description of the collection of these data.

Taking this a step further, this SCO system was also tested as a quantitative sensor for another solvent. Exposure of either the dry compound **1**, or the THF solvated **1**·THF, to CHCl_3_ vapours for 24 hours results in the CHCl_3_ solvated analogue **1**·CHCl_3_ (confirmed by CHN and TGA analysis, see ESI†), for which the *T*_1/2_ is 248 K. Interestingly, for CHCl_3_ the *T*_1/2_ dependence of the SCO event on *n*(CHCl_3_) appears to‡Transfer losses between measurements precluded measurement of further cycles.be more logarithmic in nature (Fig. 6 and S26†). The relationship between *T*_1/2_ and *n*(CHCl_3_) is described by the equation:

2 *T*_1/2_ = 27.0 log_10_[*n*(CHCl_3_)] + 243

with a standard error in the slope of 1.9 log_10_[*n*(CHCl_3_)], significantly different from 0 (*p* < 0.001). Presumably this logarithmic response is because the inter-guest interactions are stronger for CHCl_3_ than for THF, and these guest–guest interactions are becoming more significant at higher loadings. This is consistent with CHCl_3_ having a higher propensity to hydrogen bond than THF. A logarithmic dependence on guest concentration could be beneficial in applications where a higher sensitivity over a narrow range is desired. Hence this SCO system is *also a quantitative sensor* for CHCl_3_.

In summary, **1**·THF shows an abrupt SCO at 255 K. Stepwise desolvation of **1**·THF, *via***1**·*n*THF where 0 < *n* < 1, to **1** (*n* = 0) is accompanied by a linear shifting of the SCO event by 43 K down to 212 K for **1**. The gain and loss of THF guest molecules is also shown to be reversible. These findings demonstrate that this *SCO active complex can act as a quantitative* sensor. Furthermore, the THF of solvation could be exchanged by CHCl_3_, by exposure to CHCl_3_ vapours for 24 hours. The mole fraction of CHCl_3_ guest molecules present also predictably tunes the *T*_1/2_, with it decreasing by 36 K from **1**·CHCl_3_ to **1**, but with a logarithmic relationship. Finally, it is important to note that the ability of spin crossover systems to carry out robust, reversible and quantitative solvent sensing is unlikely to be limited to **1**. Hence a wide range of both new and previously reported compounds, both discrete and polymeric, both porous and non-porous should be investigated for their sensor potential.

## Supplementary Material

Supplementary information

Crystal structure data
