# Supplementary material for: Reversible quantitative guest sensing via spin crossover of an iron(ii) triazole
Source: Chem Sci. 2016 Feb 9;7(4):2501–5. doi: 10.1039/c5sc04583e (PMC5477032; doi:10.1039/c5sc04583e)
Supplement: Supplementary file 1 [file SC-007-C5SC04583E-s001.pdf]

Electronic Supplementary Material (ESI) for Chemical Science.
This journal is © The Royal Society of Chemistry 2016

Electronic Supporting Information for

Reversible quantitative guest sensing via spin crossover of an iron(II) triazole

Reece G. Miller and Sally Brooker*

Department of Chemistry and MacDiarmid Institute for Advanced Materials and Nanotechnology, University of Otago, PO Box 56, Dunedin, 9054, New Zealand

Fax: +64 3 4797906

Tel: +64 3 4797919

E-mail: sbrooker@chemistry.otago.ac.nz

General remarks regarding organic synthesis

N-(p-tolyl)-benzenethioamide[1] and pyrazine-2-carbohyrdazide[2] were prepared as previously described. Dry ethanol was prepared by distilling absolute ethanol from Mg/I_2. Other chemicals were bought commercially and used as received.

Synthesis of tolpzph

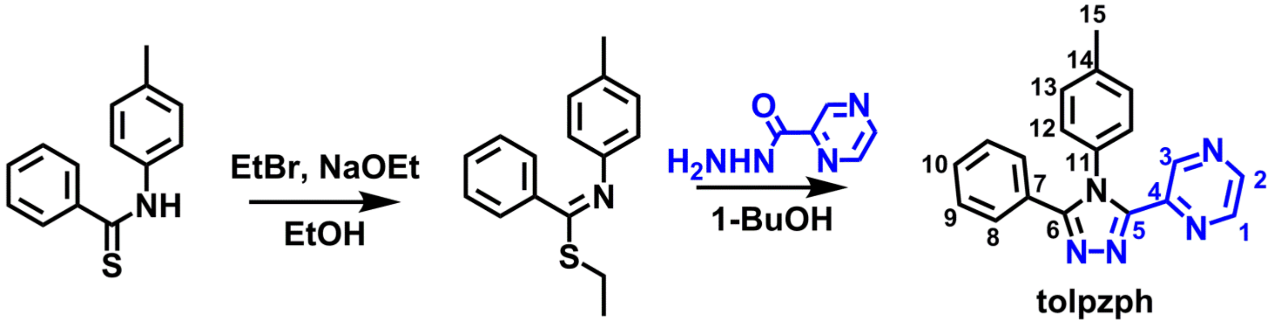

***Figure S1.** Synthesis of 4-(p-tolyl)-3-phenyl-5-pyrazyl-1,2,4-triazole (tolpzph).*

Sodium (46 mg, 2.0 mmol) was dissolved in dry EtOH (5 mL) before bromoethane (120 mg, 1.09 mmol) and *N*-(p-tolyl)-benzenethioamide (250 mg, 1.09 mmol) were added. The brown solution was stirred at 60 °C for 6 hours, during which time a white precipitate formed, and then allowed to cool to ambient temperature. The suspension was filtered to remove NaBr, then the solvent was removed at reduced pressure to give a brown oil. This was dissolved in DCM (50 mL) and washed with water (3 x 50 mL) and saturated aqueous NaCl solution (50 mL), then dried over Na_2SO_4 before being taken to dryness at reduced pressure to give the ethylated thioamide as a yellow oil. The crude oil was dissolved in n-BuOH (5 mL), solid pyrazine-2-carbohydrazide (135 mg, 0.98 mmol) was added and the mixture heated to 130 °C, at which point the suspension cleared to give a golden solution. Heating was continued for 4 days, over which time the solvent almost completely evaporated. The resulting slurry was allowed to cool to ambient temperature and then dissolved in $CHCl_3$ (75 mL) and washed with H_2O (3 x 25 mL). Then the organic layer was taken to dryness to give an off-white solid which was suspended in Et_2O (10 mL) and sonicated, then filtered off, and washed with Et_2O (2 x 20 mL) to yield pure 4-(p-tolyl)-3-phenyl-5-pyrazyl-1,2,4-triazole (**tolpzph**) as an off-white solid (266 mg, 0.85 mmol, 78% based on *N*-(p-tolyl)-benzenethioamide). Elemental analysis calc. for $C_{19}H_{15}N_5$ (313.3 g mol^{-1}): C 72.83 H 4.82 N 22.35; found: C 72.91 H 4.73 N 22.41%. HR-ESI-MS (pos.) *m/z* = 336.1217 calc. for $[C_{19}H_{15}N_5Na]^+$ = 336.1220; *m/z* = 314.1405 calc. for $[C_{19}H_{16}N_5]^+$ = 314.1400. ^{1}H NMR (400 MHz, $CDCl_3$): δ (ppm) 9.33 (1H, s, H3), 8.50 (1H, d, JH1-H2 = 2.5 Hz, H1), 8.33 (1H, d, JH1-H2 = 2.5 Hz, H2), 7.47 (2H, d, JH9-H8 = 7.2Hz, 2xH9), 7.37 (1H, t, JH10-H9 = 7.3Hz, H10), 7.29 (2H, m, $W_{h/2}$ = 26 Hz 2xH8), 7.19 (2H, d, JH13-H12 = 8.1 Hz, 2xH13), 7.10 (2H, d, JH12-H13 = 8.1Hz, 2xH12), 2.41 (3H, s, 3xH15). ^{13}C NMR (100 MHz, $CDCl_3$): δ (ppm) 156.14 (C7), 151.61 (C4), 145.28 (C3), 144.41 (C1), 143.29 (C2), 143.26 (C5), 139.58 (C14), 132.58 (C11), 129.99 (C13), 129.87 (C10), 128.79 (C8), 128.43 (C9), 127.60 (C12), 126.53 (C6), 21.29 (C15).

NMR spectra of tolpzph

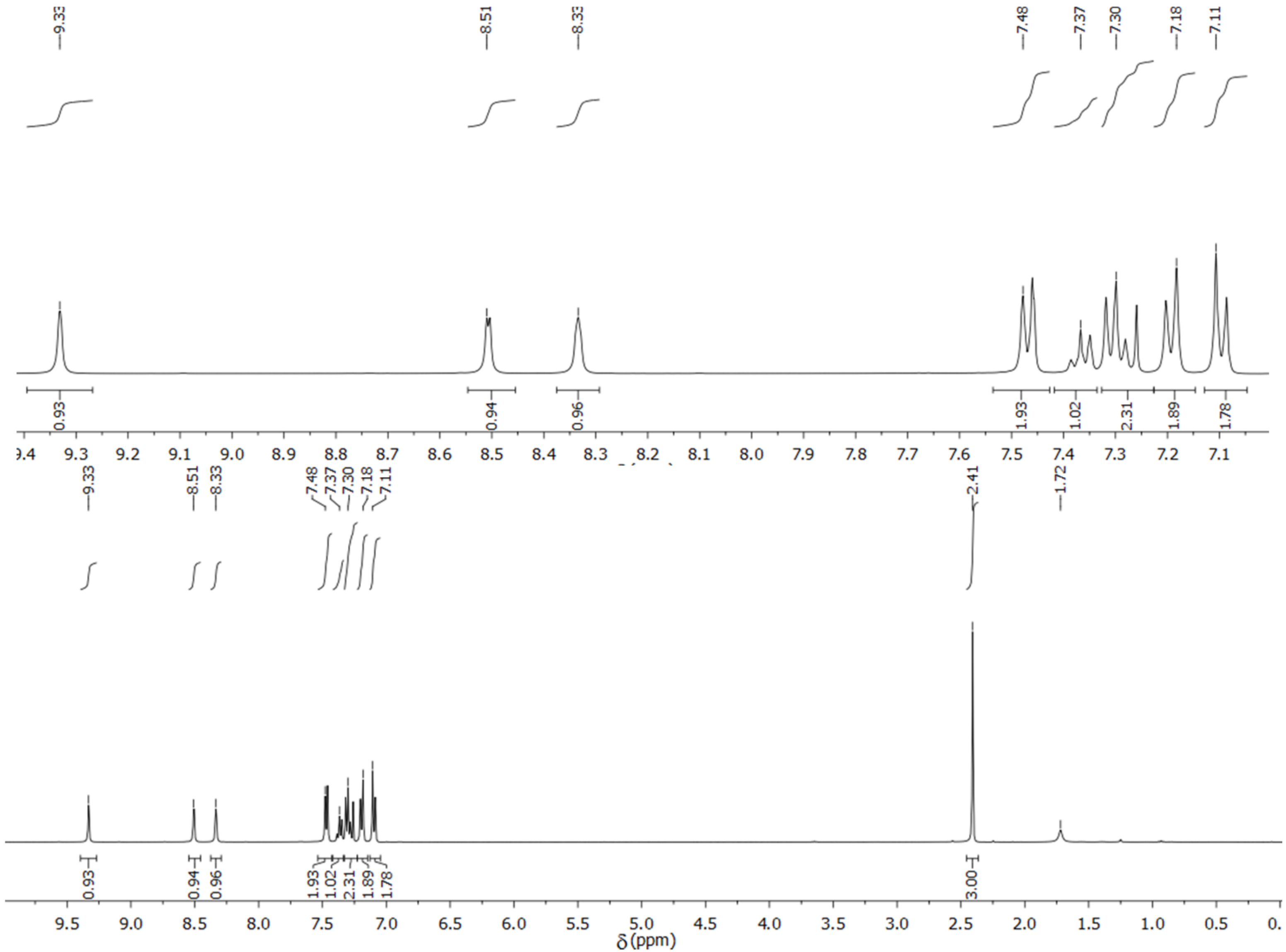

Figure S2. *^{1}H NMR spectra of tolpzph (400 MHz, $CDCl_3$, 298 K).Top:expansion of the aromatic region. Bottom: full range.*

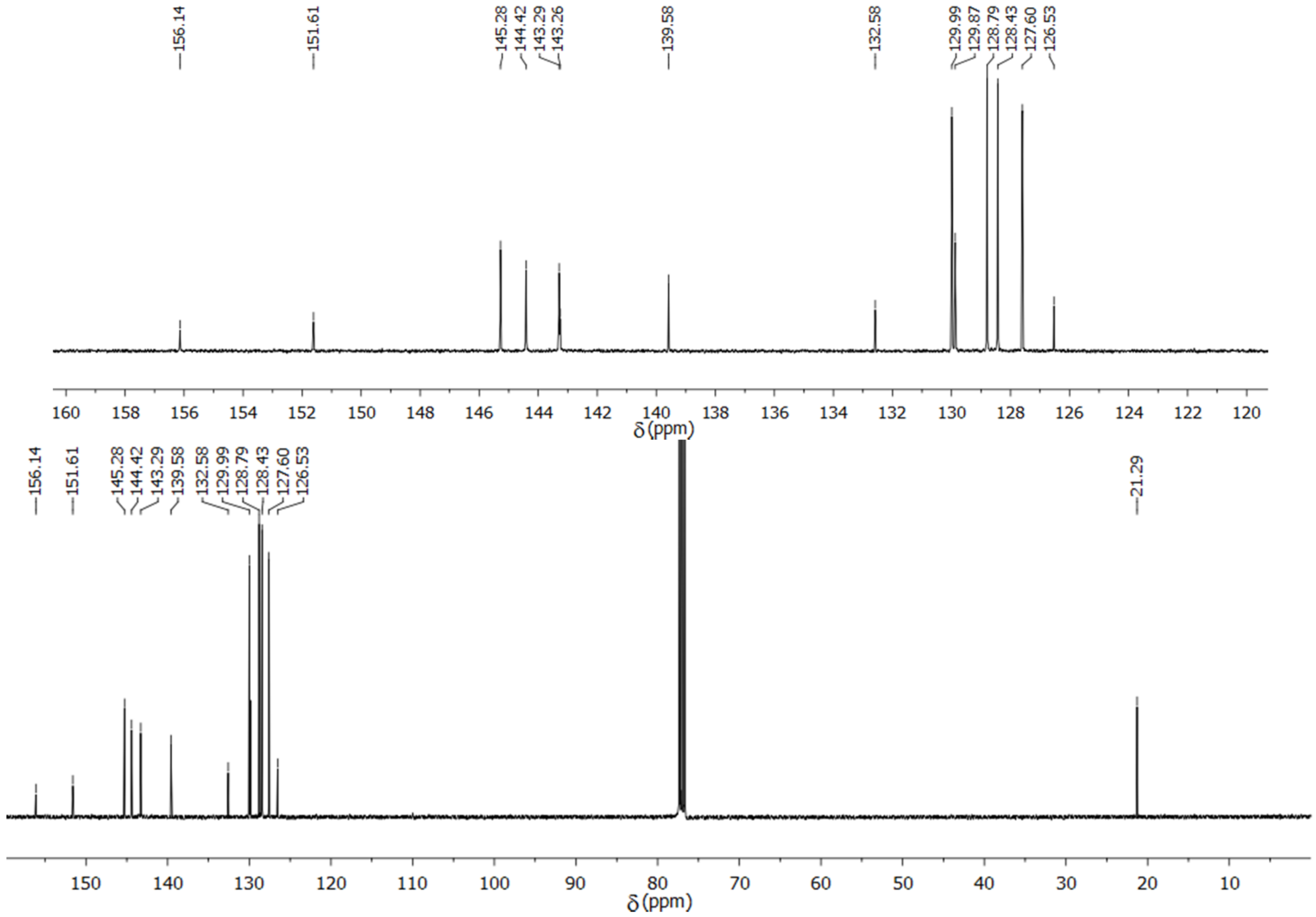

***Figure S3**. ^{13}C NMR spectra of tolpzph (100 MHz, $CDCl_3$, 298 K).Top:expansion of the aromatic region. Bottom: full range.*

Complex synthesis

General remarks regarding complex synthesis

Dry THF and dry Et_2O were obtained from a Pure Solv MD-6 Solvent Purification System and degassed by bubbling with dry $Ar_{(g)}$ for 20 min before use. Other chemicals were bought commercially and used as received.

[Fe^{II}(tolpzph)$_2$(NCS)$_2$]·THF (1·THF)

Method A (microcrystals). *Under standard Schlenk conditions,* a yellow solution of anhydrous $FeCl_2$ (16.2 mg, 0.127 mmol) in dry THF (10 mL) was added via cannula to solid NaNCS (21.0 mg, 0.255 mmol) and the mixture stirred for 20 minutes causing the solution to turn deep red and a white precipitate to form. The suspension was filtered and the deep red filtrate transferred via cannula to a colourless solution of **tolpzph** (80 mg, 0.255 mmol) in dry THF (5 mL), resulting in a violet solution. The solution was stirred for 30 minutes, then reduced to half the volume by bubbling with $N_{2(g)}$. On standing for 48 hours at room temperature dark green microcrystals precipitated. These were filtered and surface dried by repeated brief vacuum and refill (dry $N_{2(g)}$) cycles (x5) on the Schlenk line (82 mg, 94.3 µmol, 74%). These green microcrystals were stored under $Ar_{(g)}$ at -18 °C as a precaution. Elemental analysis calc. for **1**·THF, $C_{44}H_{38}N_{12}O_1S_2Fe$ (870.8 g mol^{-1}): C 60.69, H 4.40, N 19.30, S 7.36; found: C 60.38, H 4.78, N 18.92, S 7.20%. TGA Δmass (at 403 K, after 30 min): 8.60 %, calc. for ·THF (78.1 g mol^{-1}): 8.96% HR-ESI-MS (pos.): *m/z* = 314.1372, calc. for [H(**tolpzph**)]$^+$: 314.1400; 336.1181, calc. for [Na(**tolphph**)]$^+$: 336.1200; 649.2474 calc. for [Na(**tolpzph**)$_2$]$^+$: 649.2547. IR (ATR, *inter alia*): 2106 (w, N≡C stretch for LS), 2064 (vs, N≡C stretch for HS), 1510 (m), 1481 (s), 1464 (m), 1438 (m), 1384 (w), 1180 (w), 1140 (w), 1054 (w), 1030 (m), 821 (w), 780 (m), 698 (m), 726 (m), 698 (s), 613 (s), 522 (w), 407 (w) cm^{-1}.

Method B (single crystals). *Under standard Schlenk conditions,* a yellow solution of anhydrous $FeCl_2$ (24.3 mg, 0.192 mmol) in dry THF (5 mL) was added via cannula to a colourless solution of NaNCS (31.2 mg, 0.384 mmol) in dry THF (5 mL) and the mixture stirred for 20 minutes causing the solution to turn deep red and a white precipitate to form. The suspension was filtered and the deep red filtrate transferred via cannula to a colourless solution of **tolpzph** (120 mg, 0.384 mmol) in dry THF (15 mL), resulting in a violet solution. The solution was stirred for 12 hours under an $Ar_{(g)}$ balloon, then transferred via cannula to an H-tube and subjected to vapour diffusion of dry, degassed Et_2O (40 mL). After 1 week at room temperature, dark green, rod shaped, single crystals of **1**·THF suitable for X-ray crystallography formed. These were filtered off and dried by repeated brief vacuum and refill (dry $N_{2(g)}$) cycles (x5) on the Schlenk line (51 mg, 58.6 µmol, 31%). These crystals were stored under $Ar_{(g)}$ at -18 °C as a precaution. Elemental analysis calc. for **1**·THF, $C_{44}H_{38}N_{12}O_1S_2Fe$ (870.8 g mol^{-1}): C 60.69, H 4.40, N 19.30, S 7.36; found: C 60.28, H 4.58, N 18.72, S 7.29% HR-ESI-MS (pos.): *m/z* = 314.1372, calc. for [H(**tolpzph**)]$^+$: 314.1400; 336.1181, calc. for [Na(**tolphph**)]$^+$: 336.1200; 649.2474 calc. for [Na(**tolpzph**)$_2$]$^+$: 649.2547. IR (ATR, *inter alia*): 2106 (w, N≡C stretch for LS), 2064 (vs, N≡C stretch for HS), 1510 (m), 1481 (s), 1464 (m), 1438 (m), 1384 (w), 1180 (w), 1140 (w), 1054 (w), 1030 (m), 821 (w), 780 (m), 698 (m), 726 (m), 698 (s), 613 (s), 522 (w), 407 (w) cm^{-1}.

Additional information regarding solvent content of samples obtained as described above. When drying the compound using repeated brief vacuum and refill (dry $N_{2(g)}$) cycles it is possible to remove some of the solvent of crystallisation. This can be avoided if sufficient care is taken. For three batches of **1**·THF: TGA Δmass (at 403 K, after 30 min): 8.60 %, 9.31 % and 8.27 % calc. for ·THF (78.1 g mol^{-1}): 8.96%.

However, when a sample was instead kept under vacuum for approximately 10 min, TGA gave a Δmass (at 403 K, after 30 min): 6.63 % calc. for 0·75THF (78.1 g mol^{-1}): 6.72%. Please note that if a sample is excessively dried like this, then exposure to THF vapour for 2 hours, followed by 30 minute drying in air, can be used to re-solvate it and obtain **1**·THF.

$[Fe^{II}(tolpzph)_2(NCS)_2]\cdot CHCl_3$ (1·$CHCl_3$)

A sample of **1**·THF (30 mg) was placed in a small sample tube (~5 cm^3). This sample tube was then placed in a large sample tube (~30 cm^3) containing $CHCl_3$ (5 mL) and the large vial capped. After 24 hours of vapour diffusion the THF of solvation had been replaced by $CHCl_3$. Samples were then left exposed to air for 30 min before use in fractional solvent content experiments as described on page S7, or microanalysis and TGA, in order to allow any excess surface adsorbed solvent to evaporate. As there was only minimal surface solvent (for this vapour method, as compared to the samples isolated from solution), this air-drying was sufficient to ensure the sample dried without water damage as a result of the solvent becoming hydrated. Elemental analysis calc. for **1**·$CHCl_3$, $C_{41}H_{31}N_{12}Cl_3S_2Fe$ (918.1 g mol^{-1}): C 53.64, H 3.40, N 18.31, S 6.98, Cl 11.58; found C 53.04, H 3.30, N 17.85, S 7.02 Cl 11.60% TGA Δmass (at 403 K, after 30 min, for four different preparations): 11.91 %, 12.51 %, 13.58 %, 13.90 %, calc. for ·$CHCl_3$ (119.4 g mol^{-1}): 13.01 %

Details of instrumentation and techniques

Elemental analyses were measured at the Campbell Micro-analytical Laboratory, University of Otago. IR spectra were recorded on a Bruker ATR-IR spectrometer with an Alpha-P module. ^{1}H and ^{13}C NMR spectra were recorded on a 400 MHz Varian 400MR spectrometer at 298 K. Chemical shifts are referenced to residual solvent peaks ($CDCl_3$: ^{1}H 7.26, ^{13}C 77.16 ppm). ESI mass spectra were recorded on a Bruker micrOTOF$_Q$ mass spectrometer in MeOH.

X-Ray crystallographic data for were collected using graphite monochromated Cu Kα radiation on an Oxford Diffraction SuperNova diffractometer equipped with a Cryostream N_2 open-flow cooling device,[3] at 100 and 273 K. Series of scans were performed in such a way as to collect a complete set of unique reflections to a maximum resolution of 0.80 Å. Raw frame data (including data reduction, inter-frame scaling, unit cell refinement and absorption corrections[4]) for all structures were processed using CrysAlis Pro.[5] Structures were solved using SHELXS-2014[6] and refined using full-matrix least-squares on F^2 within the X-Seed graphical user interface.[7] All non-hydrogen atoms were refined with anisotropic displacement parameters. Hydrogen atoms were restrained in calculated positions using a riding model. Crystallographic data for the structures have been deposited with the Cambridge Crystallographic Data Centre, CCDC 1434103-1434104.

TGA data were collected on a TA Instruments TGA Q_{50}. In each case the temperature was ramped at 1 K min^{-1} between 298 K and 403 K and then held constant until a total elapsed time of 150 min after which the mass was essentially constant (Δmass < 1% per hour).

Solid state visible reflectance data were recorded on a Perkin Elmer Lambda 950 UV/Vis/NIR spectrometer either in the range 400-1500 nm or in the range 400-800 nm, as indicated. Samples were attached to a solid support of Labsphere reflectance standard using double-sided tape (Sellotape® Double-Sided). Strong absorbance of this tape precluded measurement below 400 nm. See Figure .

Variable temperature magnetic susceptibility measurements were carried out in house on a Quantum Design Versalab, Cryogen-free PPMS susceptometer equipped with a vibrating sample mount (VSM) in the temperature range 50-400 K. Data were corrected for the diamagnetism of the sample and a background correction for the sample holder was applied. The data were collected as indicated for each experiment, in either (a) 'settle mode' (5 K min^{-1} between data points which were recorded 1 min after the temperature was within the smaller value of $\pm$0.5 K or $\pm$ 0.5% of the target value) or (b) 'sweep mode' (scan rate of 1 K min^{-1} with data collected continuously). These data were also corrected for temperature lag between the sample and instrument thermometer. This was done by measuring the temperature difference between the heating and cooling runs (outside of the range of the SCO event) that gave the same magnetic susceptibility, and assigning the mean temperature to this susceptibility whilst also noting the temperature correction. Doing this at several different places resulted in a constant temperature correction so this was applied across the 50-300 K range.

Full methodology for determining the relationship between fractional solvent content and spin crossover behaviour: Samples weighing between 14 mg and 20 mg (2 samples for $CHCl_3$, 6 samples for THF) were loaded into a PPMS sample holder and $\chi_M T$ measured in the Versalab PPMS over the range 280→200→280 K at a scan rate of 1 K min^{-1}. The average of the temperature at which $\chi_M T$ = 1.5 cm^3 mol^{-1} K in the cooling and heating directions was recorded and then a small sample (2-4 mg) was removed from the PPMS holder for TGA to determine the corresponding mass loss on heating it (see above) and therefore solvent content. The remaining sample in the PPMS holder was then returned to the Versalab PPMS and heated in the range 280→400→280 K at scan rates ranging from 1-5 K min^{-1} and with isothermal periods at 400 K in the range 0-120 min. The variation in time spent at 400 K was used to remove differing amounts of solvent from the sample in order to provide a wide range of data points. After this heating cycle $\chi_M T$ was again measured in the range 280→200→280 K at a scan rate of 1 K min^{-1}, then another small sample (2-4 mg) taken for TGA. This process was then repeated for a total of 4 or 5 cycles per 14-20 mg sample (until there was no sample left). The molecular weight used in the data analysis was always that for **1**·THF (870.8 g mol^{-1}).

Refinement details for 1·THF: A crystal was mounted and a dataset collected at 273 K (mostly HS), then the temperature was dropped to 100 K (mostly LS) and a second dataset collected on the same crystal. At both temperatures there is one half of the complex and one half THF in the asymmetric unit (and two complexes and two THF molecules in the unit cell). The THF is disordered over two overlapping quarter occupancy sites, with all four carbon atoms common to

both positions (hence half occupancy). PART -1 contains all four half occupancy C atoms and one quarter occupancy O atom (O35), whilst PART -2 contains the other one quarter occupancy O atom (O45). H atoms were not included on this disordered THF. DFIX was used to restrain the disordered THF geometries. Unsurprisngly, the U values of this THF were far better at 100 K than at 273 K.

Solid state structures of [Fe^{II}(tolpzph)$_2$(NCS)$_2$]·THF (**1**·THF)

Table S1. Crystallographic data for **1**·THF at 100 K and 273 K (same crystal, 273 K collected first)

	[Fe(**tolpzph**)$_2$(NCS)$_2$]·THF at 100 K	[Fe(**tolpzph**)$_2$(NCS)$_2$]·THF at 273 K
Empirical formula	$C_{44}H_{38}FeN_{12}OS_2$	$C_{44}H_{38}FeN_{12}OS_2$
Formula weight	870.83	870.83
Temperature (K)	100(2)	273(2)
Wavelength	1.54184	1.54184
Crystal system	Orthorhombic	Orthorhombic
Space group	*Pbca*	*Pbca*
a (Å)	7.45552(17)	7.5264(2)
b (Å)	21.1136(4)	21.5575(5)
c (Å)	26.5813(6)	26.9774(6)
α (°)	90	90
β (°)	90	90
γ (°)	90	90
Volume (Å^3)	4184.24(15)	4377.09(18)
Z	4	4
Density (calculated, in Mg/m^3)	1.382	1.321
Absorption coefficient (mm^{-1})	4.235	4.048
F(000)	1808	1808
Crystal size (mm^3)	0.30 x 0.10 x 0.04	0.30 x 0.10 x 0.04
Theta range for data collection (°)	3.33 to 76.437	3.276 to 74.400
Index ranges	-6<=h<=9<br>-26<=k<=26<br>-33<=l<=29	-6<=h<=9<br>-26<=k<=26<br>-33<=l<=29
Reflections collected	17512	16694
Independent reflections	4369	4336
Completeness to theta (%)	99.3	99.9
Max. and min. transmission	0.471 and 0.863	0.471 and 0.863
Refinement method	Full matrix least-squares on F^2	Full matrix least-squares on F^2
Data / restraints / parameters	4369 / 17 / 305	4336 / 5 / 305
Goodness-of-fit on F^2	1.061	1.055
Final R indices [I>2sigma(I)]	R_1 = 0.0670<br>wR_2 = 0.1750	R_1 = 0.0537<br>wR_2 = 0.1617
R indices (all data)	R_1 = 0.0785<br>wR_2 = 0.1887	R_1 = 0.0671<br>wR_2 = 0.1765
Largest diff. peak and hole (e.Å^{-3})	0.97 and -0.84	0.76 and -0.84

7.1.1. Supplementary structural figures

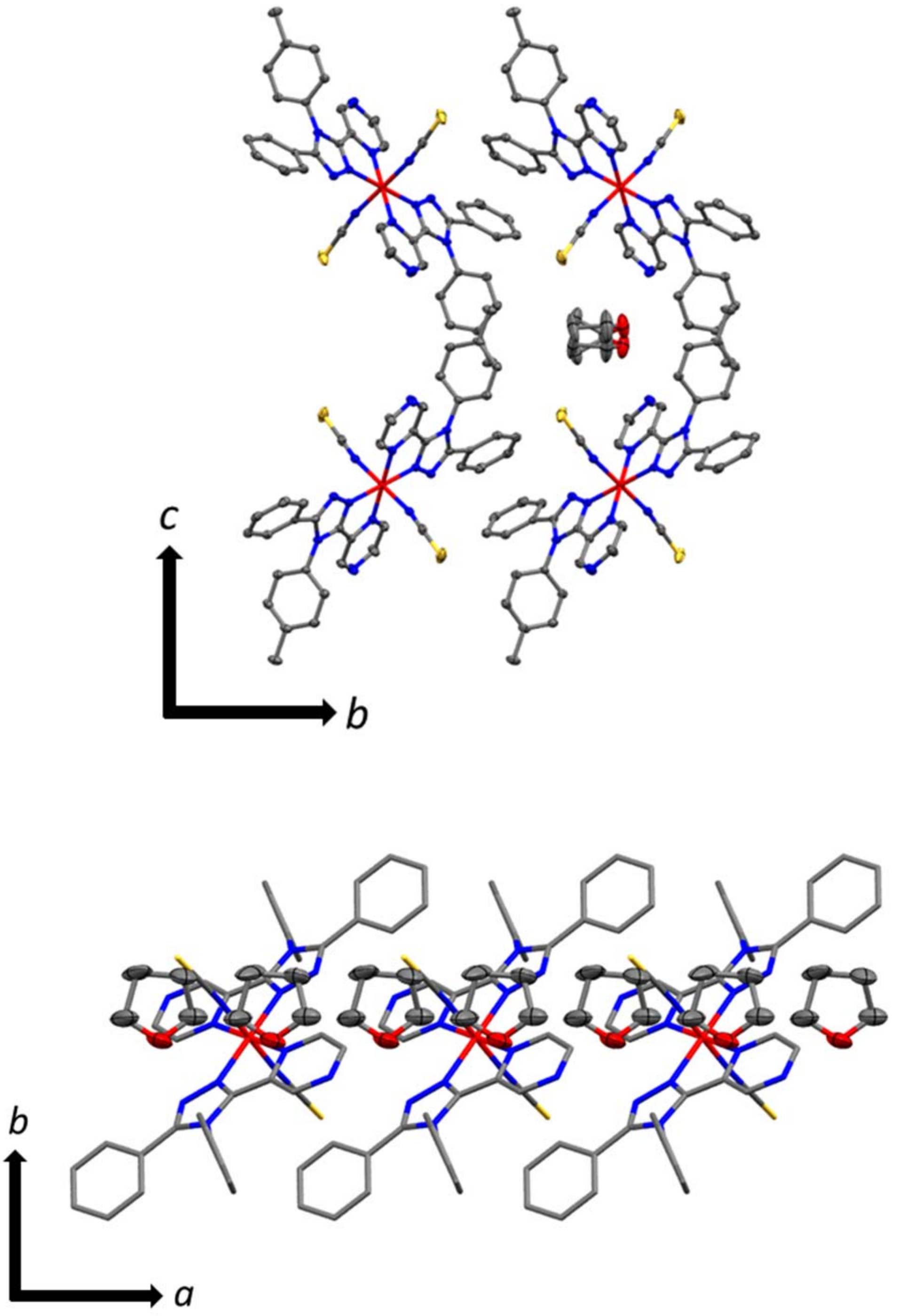

Figure S4. *Crystal packing in [Fe^{II}(**tolpzph**)$_2$(NCS)$_2$]·THF showing two of the four overlapping positions of the quarter occupancy THF molecules per complex. H-atoms have been omitted for clarity. Viewed down the a-axis (top) and c-axis (bottom).*

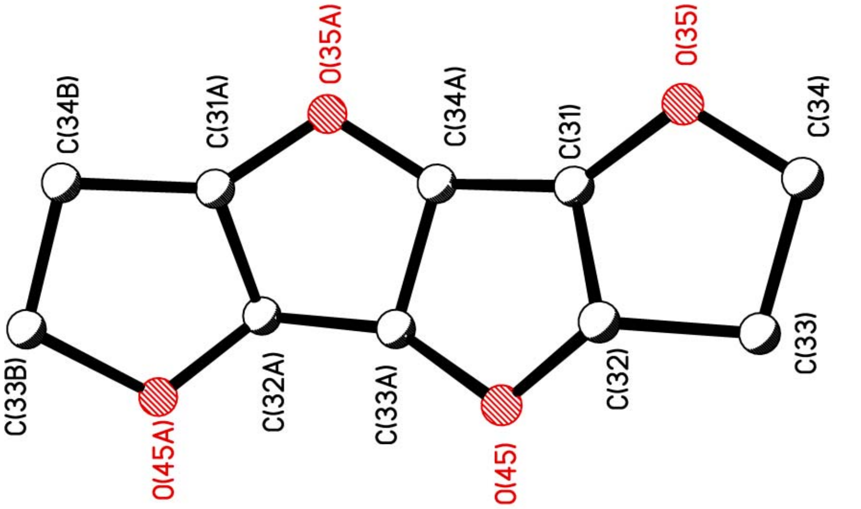

Figure S5*. Perspective view of the THF disorder in [Fe^{II}(****tolpzph****)$_2$(NCS)$_2$]·THF as viewed down the c-axis. All four ¼ occupancy positions are shown. The four carbon atoms are common to both crystallographically unique quarter occupancy components, these, and the other two quarter occupancy THF positions, are generated by a glide plane parallel to the ab-plane (translation is along the a-axis).*

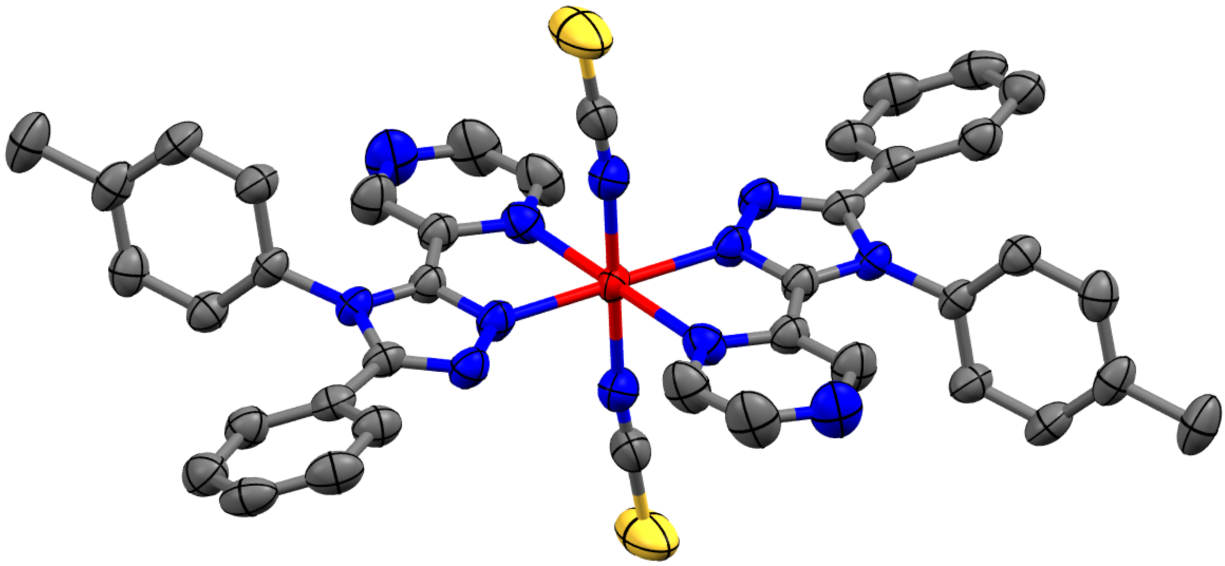

Figure S6*. Solid state structure of [Fe^{II}(tolpzph)$_2$(NCS)$_2$]·THF (****1****·THF) at 273 K. Hydrogen atoms and THF of solvation omitted for clarity, thermal ellipsoids are drawn at 50% probability.*

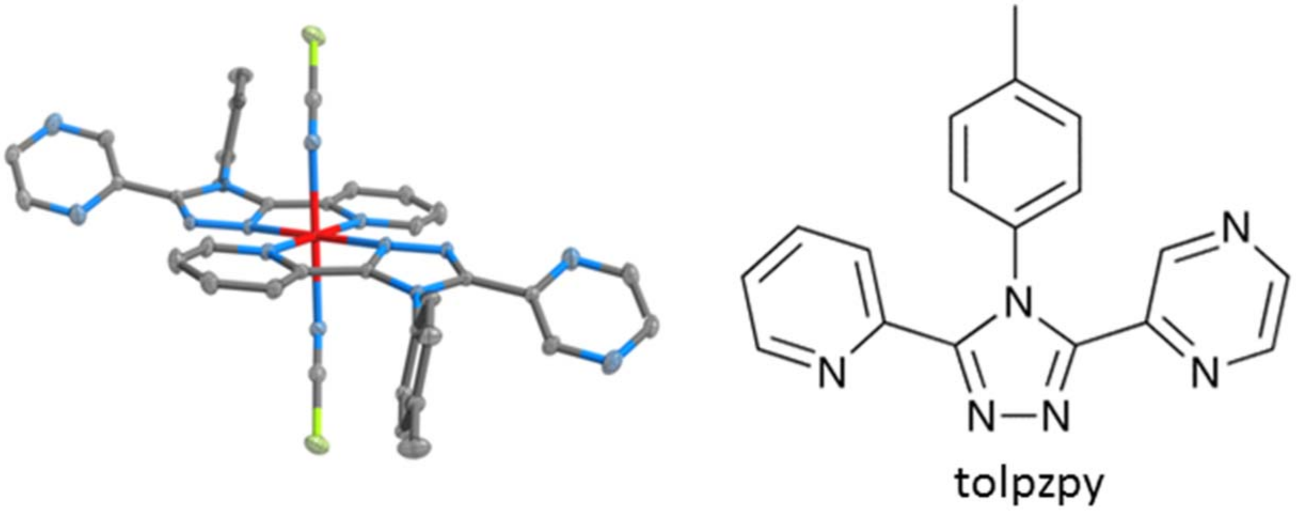

Figure S7.*Solid state structure of a previously reported, related complex [Fe^{II}(**tolpzpy**)$_2$(NCSe)$_2$]. Right: The ligand **tolpzpy**.*

IR Spectra

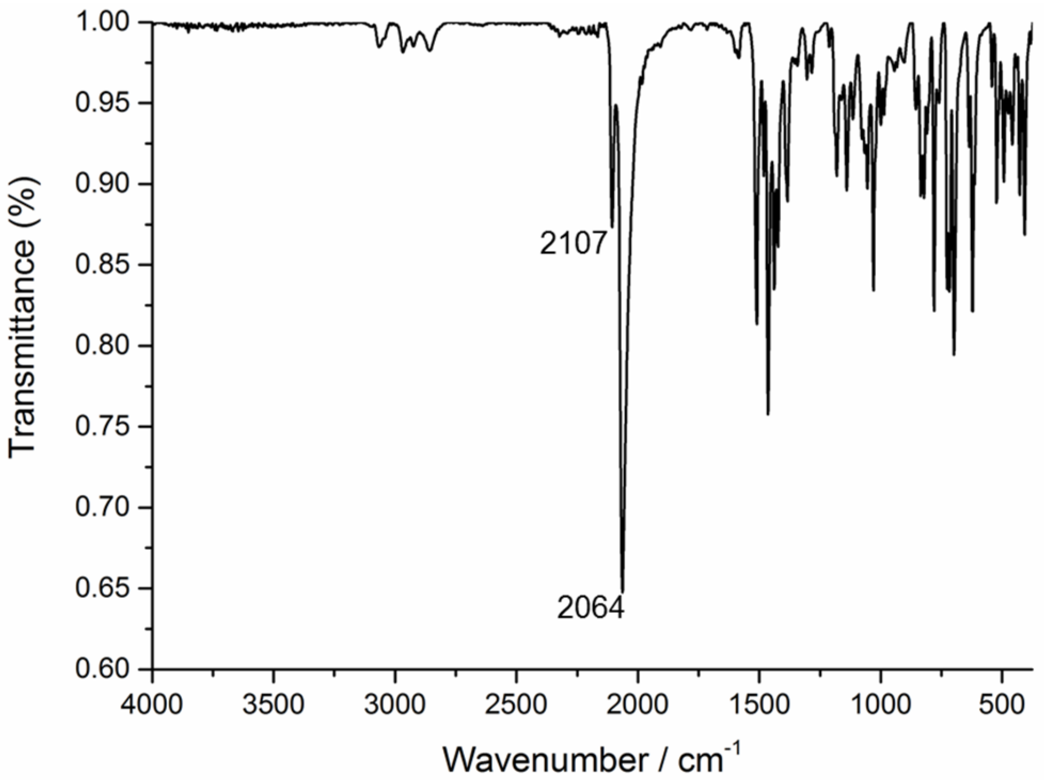

Figure S8. *Room temperature IR spectrum of as synthesised [Fe(tolpzph)$_2$(NCS)$_2$]·THF (**1**·THF).*

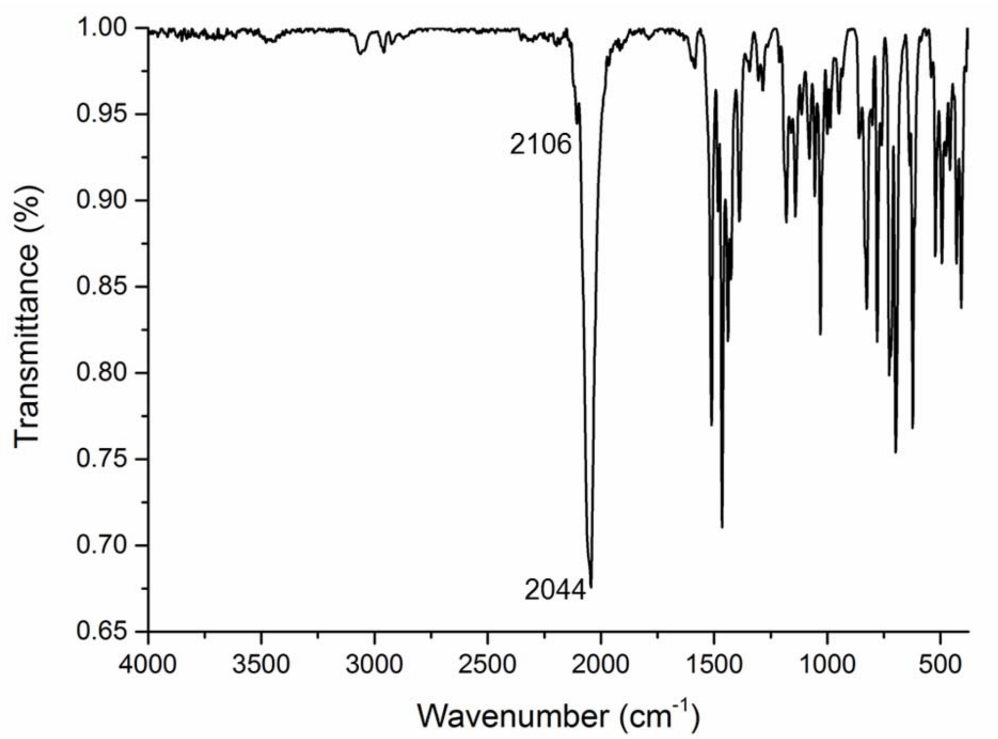

Figure S9. *Room temperature IR spectrum of [Fe(tolpzph)$_2$(NCS)$_2$] (**1**) after drying under a nitrogen flow at 130 °C for 1 h.*

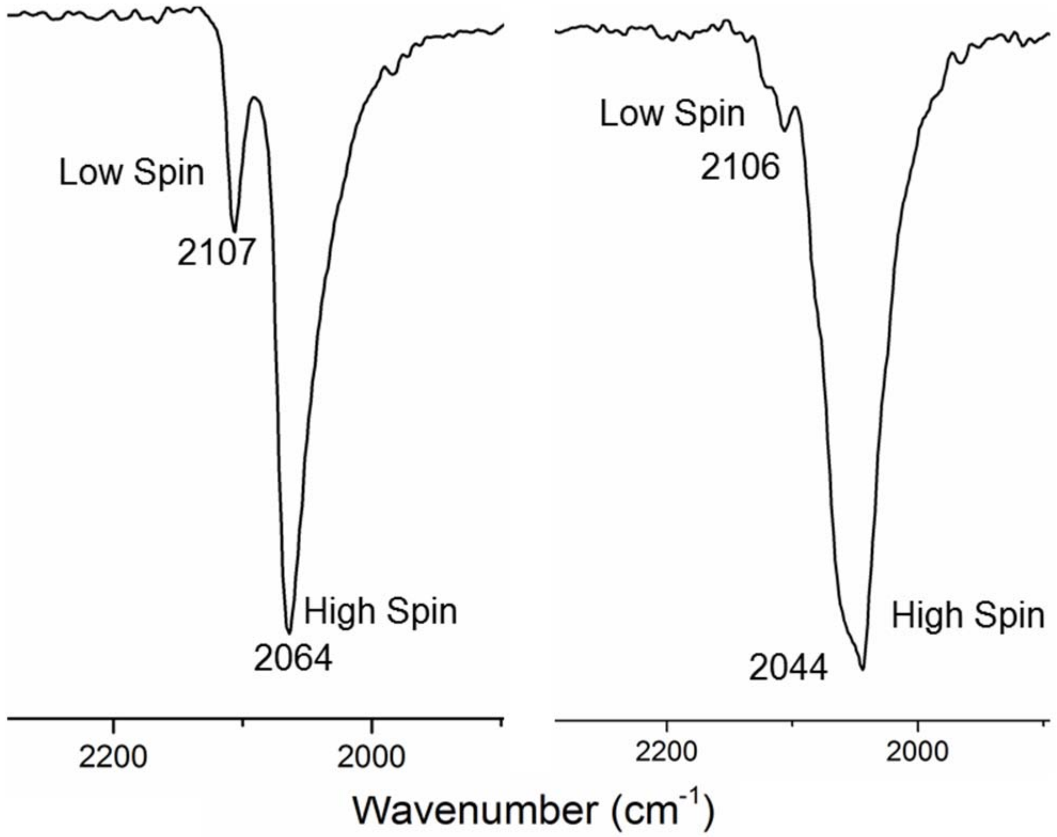

***Figure S10**. Comparison of the N≡C stretches in the room temperature IR spectra of [Fe(tolpzph)$_2$(NCS)$_2$]·THF (left) and [Fe(tolpzph)$_2$(NCS)$_2$] (right), showing a clear decrease in LS fraction on drying, consistent with a shift of the $T_{½}$ to lower temperature.*

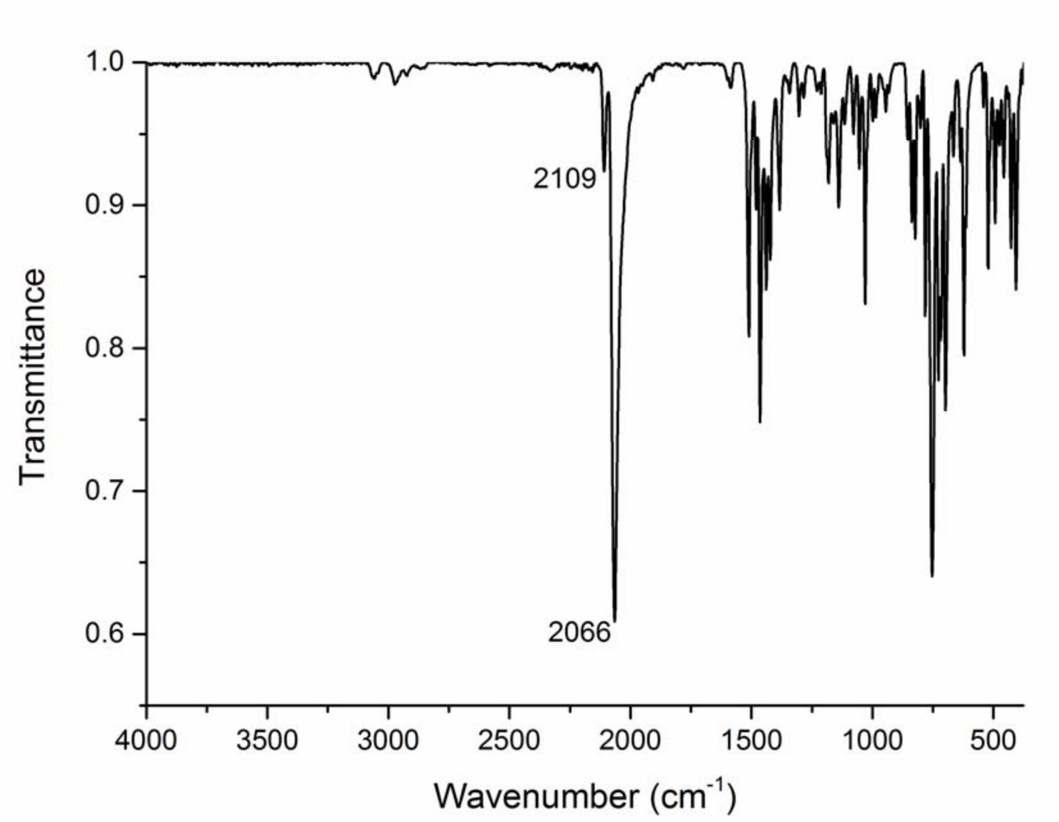

Figure S11. *IR spectrum of [Fe(tolpzph)$_2$(NCS)$_2$]·CHCl$_3$ i.e. after solvent exchange for CHCl$_3$.*

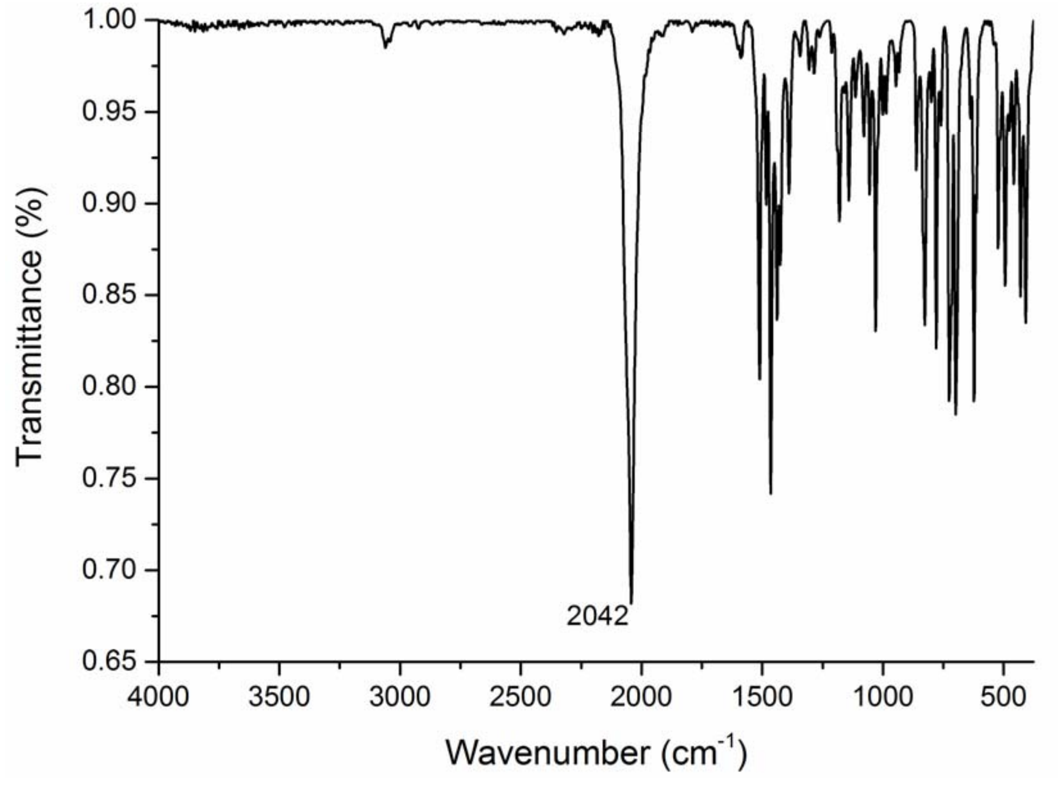

Figure S12. *IR spectrum of [Fe(tolpzph)$_2$(NCS)$_2$] after solvent exchange for CHCl$_3$ followed by drying under a nitrogen flow at 130 °C for 1 h.*

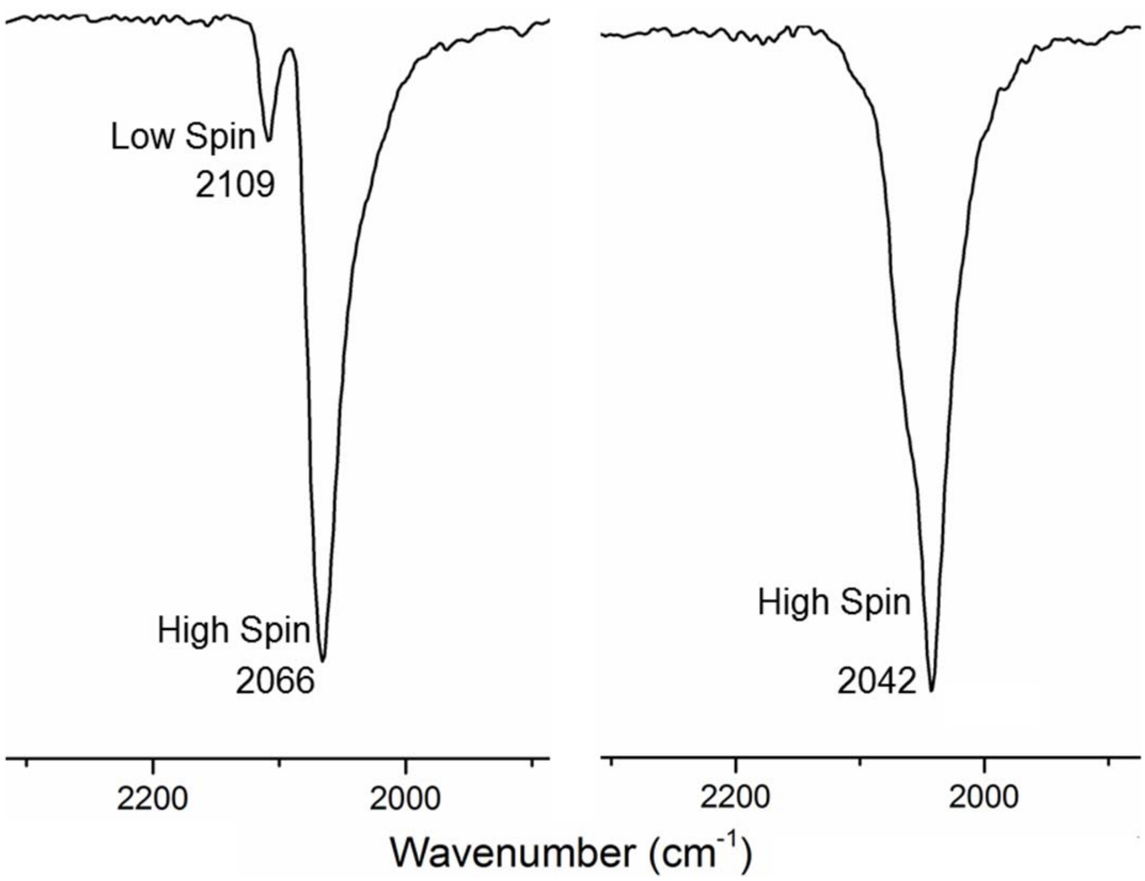

Figure S13. *Comparison of the N≡C stretches in the room temperature IR spectra of [Fe(tolpzph)*$_2$*(NCS)*$_2$*]·CHCl*$_3$ *(left) and [Fe(tolpzph)*$_2$*(NCS)*$_2$*] (right) showing a clear decrease in LS fraction on drying, consistent with a shift of the T*$_{½}$ *to lower temperature.*

Temperature dependence of the unit cell parameters for 1•THF

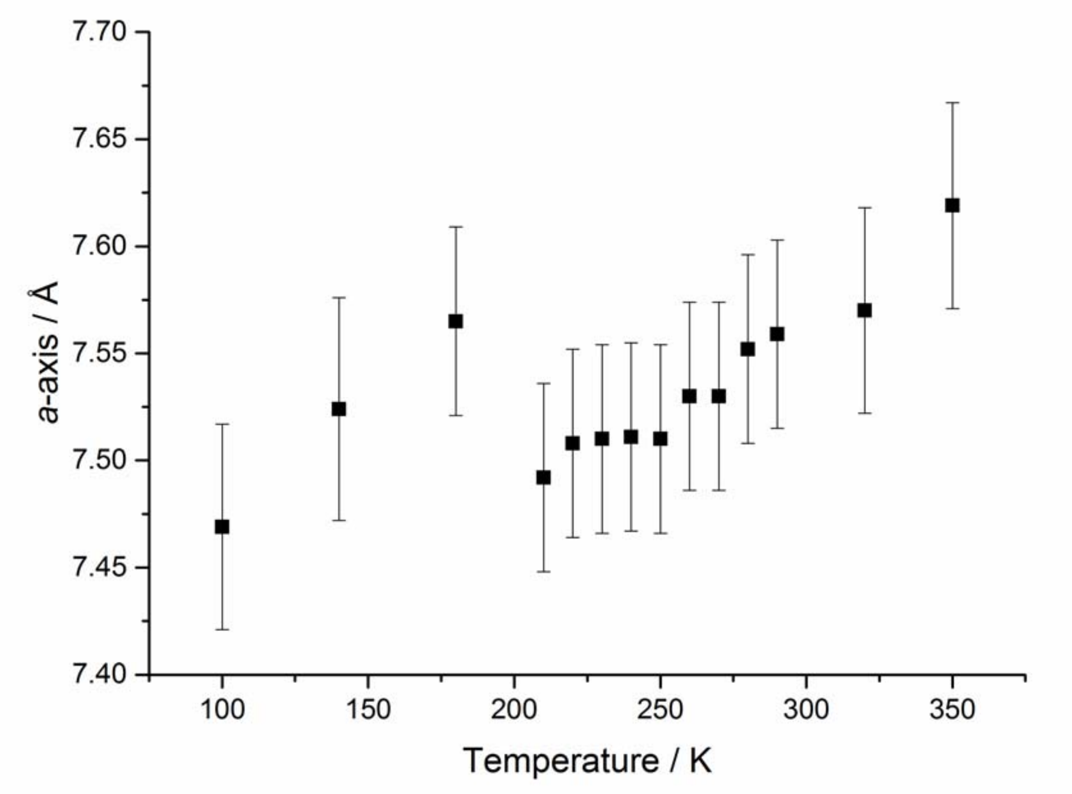

Figure S14. *Temperature dependence of the unit cell a-axis. The error bars shown represent a 95% confidence interval.*

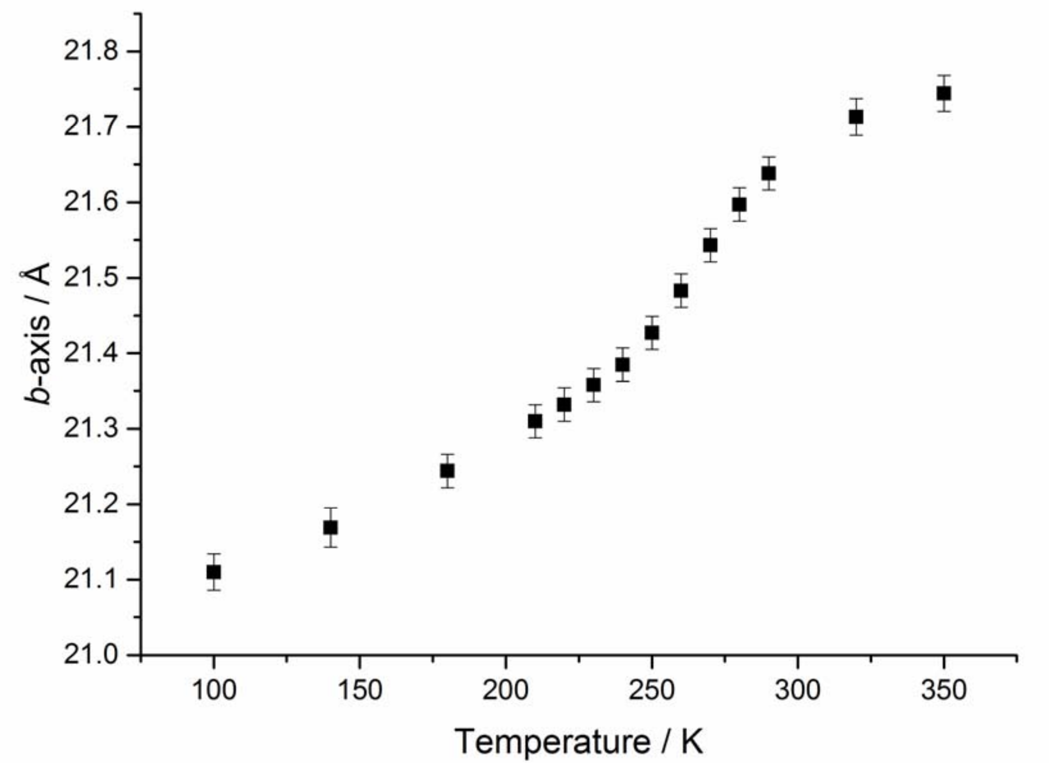

Figure S15. *Temperature dependence of the unit cell b-axis. The error bars shown represent a 95% confidence interval.*

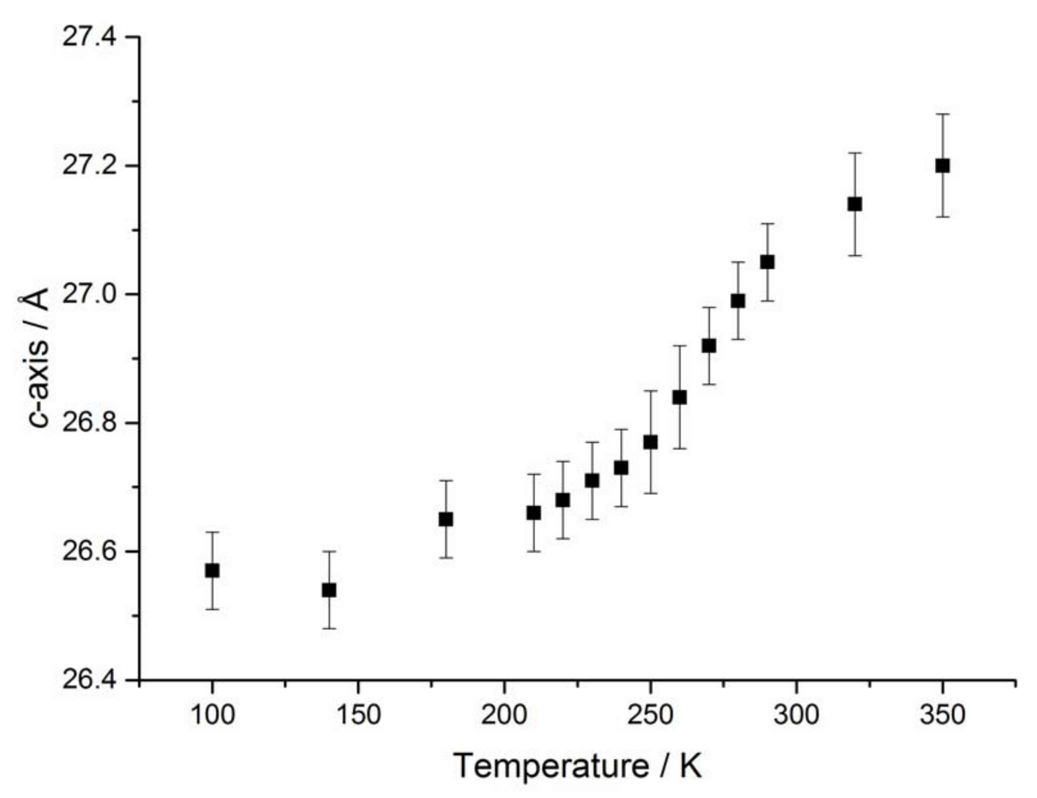

Figure S16. *Temperature dependence of the unit cell c-axis. The error bars shown represent a 95% confidence interval.*

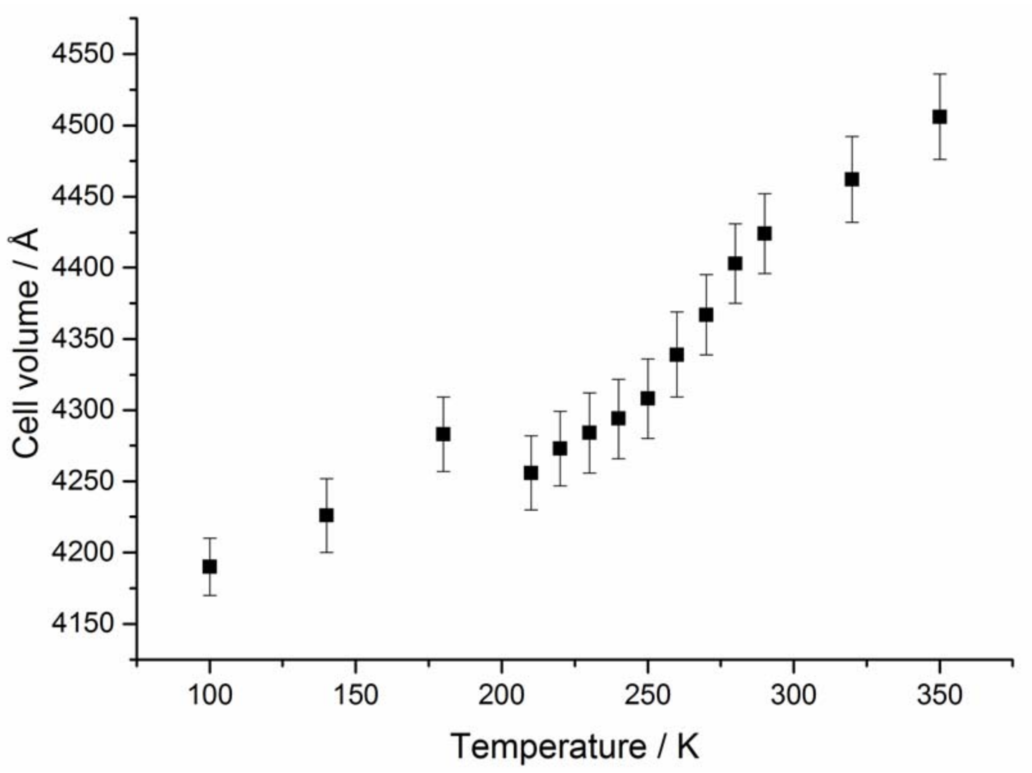

Figure S17. *Temperature dependence of the unit cell volume. The error bars shown represent a 95% confidence interval.*

Electronic absorbtion spectra

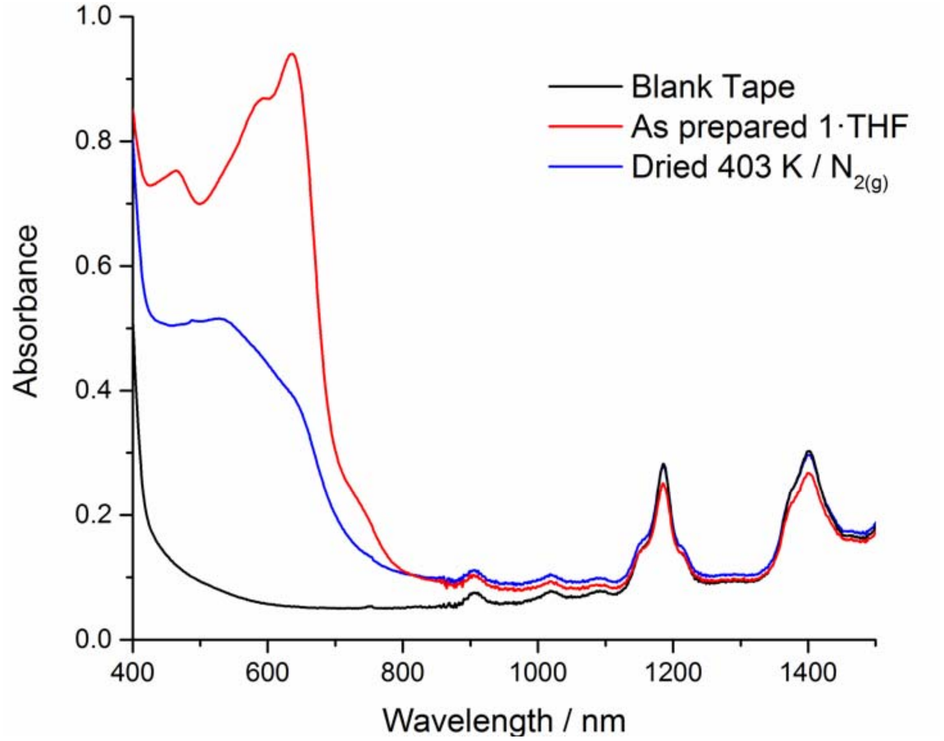

Figure S18.*Solid state reflectance UV-vis spectra of forest green* **1***·THF (as prepared) and violet* **1** *(after drying in a $N_{2(g)}$ stream at 403K for 1 h) in the range 400-1500 nm. Note: the data is presented as collected; no correction for the tape has been made.*

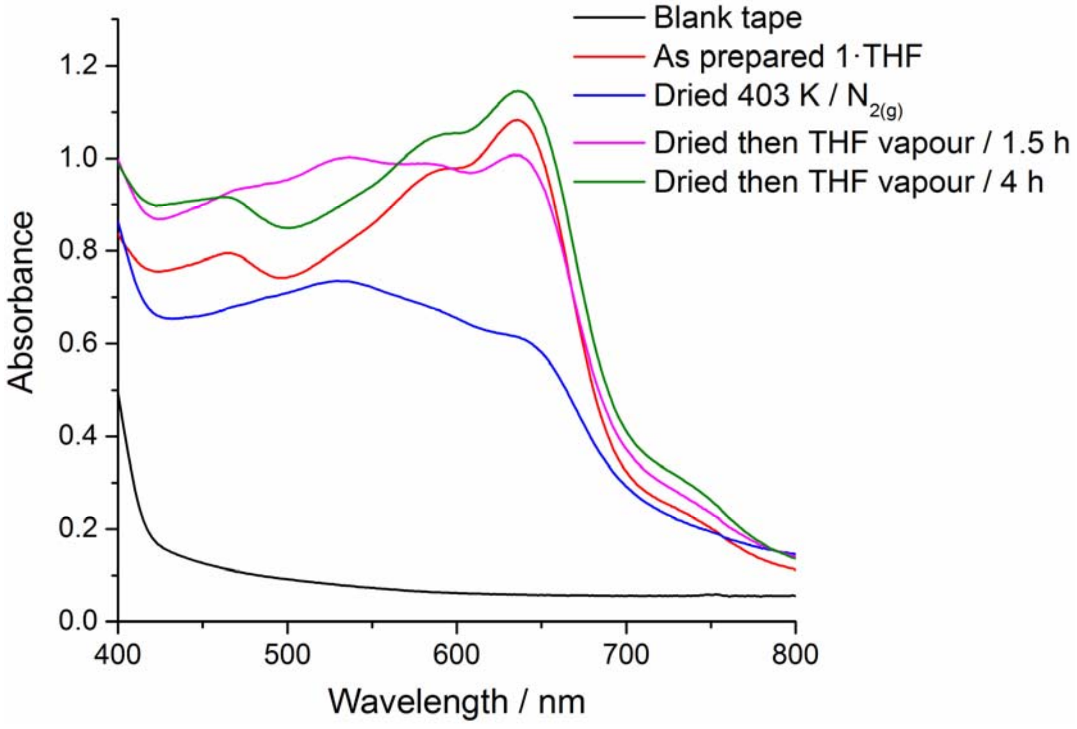

Figure S19. *Solid state reflectance UV-vis spectra of* **1***·THF (as prepared),* **1** *(after drying in a $N_{2(g)}$ stream at 403K for 1 h), and then the dried sample after exposure to THF vapour for 1.5 h, and after 4 h, in the range 400-800 nm. Note that the after 1.5 h exposure the sample was still visibly purple in parts whereas after 4 h it had turned completely green. Note: the data is presented as collected; no correction for the tape has been made.*

Thermogravimetric Analyses

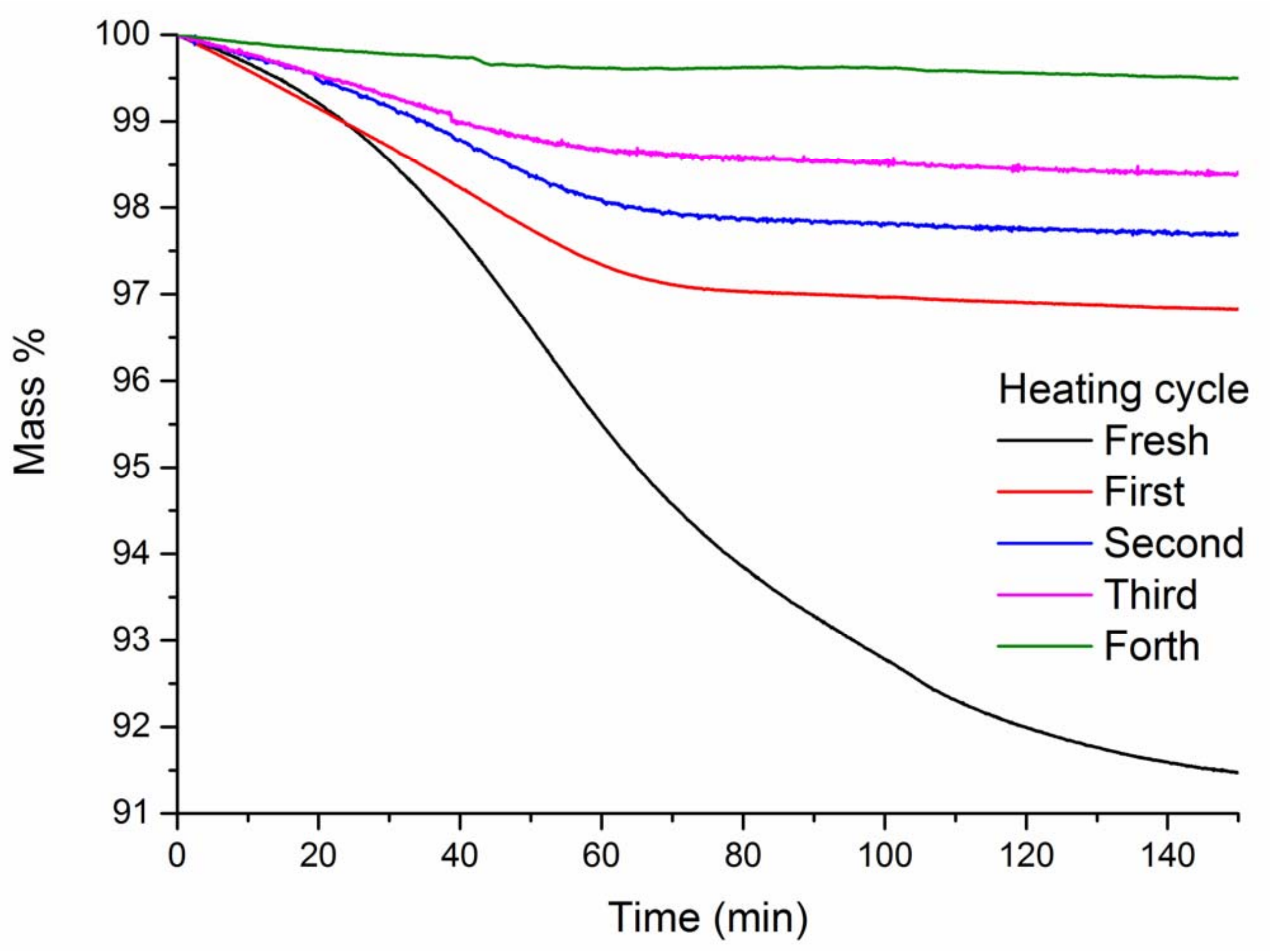

Figure S20. *Percentage mass as a function of temperature for [Fe(tolpzph)$_2$(NCS)$_2$]·THF (**1**·THF) as freshly prepared (black trace; consistent with loss of 1 THF = 8.60 %, expect 8.96 %) and then on small sample removed from the sample holder after each of 4 cycles to 400 K within the VersaLab PPMS. In each case the temperature was ramped at 1 K min^{-1} between 298 K and 403 K and then held constant until a total elapsed time of 150 min. Please note: The data after the fourth run is shown as a 400 data point (approx. 4 °C) running average to reduce noise.*

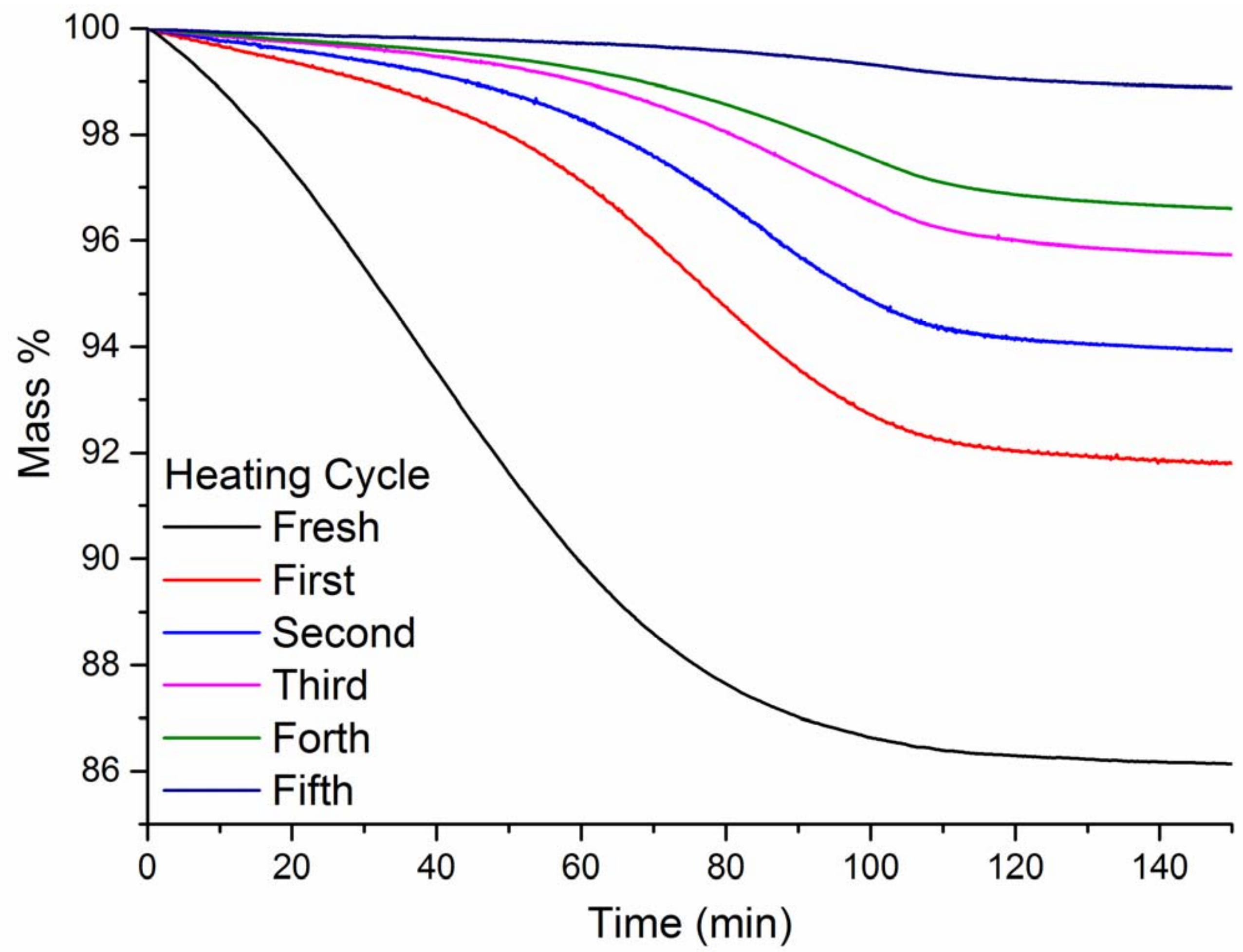

Figure S21. *Percentage mass as a function of temperature for [Fe(**tolpzph**)$_2$(NCS)$_2$]·CHCl$_3$ (**1**·CHCl$_3$) fresh after solvent exchange (THF→CHCl$_3$) (black trace; consistent with loss of 1 CHCl$_3$ = 13.85 %, expect 13.01 %) and then after repeated cycles to 400 K within the VersaLab PPMS. In each case the temperature was ramped at 1 K min^{-1} between 298 K and 403 K and then held constant until a total elapsed time of 150 min. Please note: the sample was held at 400 K in the Versalab for a period of 1 h on the fifth magnetic cycle.*

Supplementary Magnetic Data

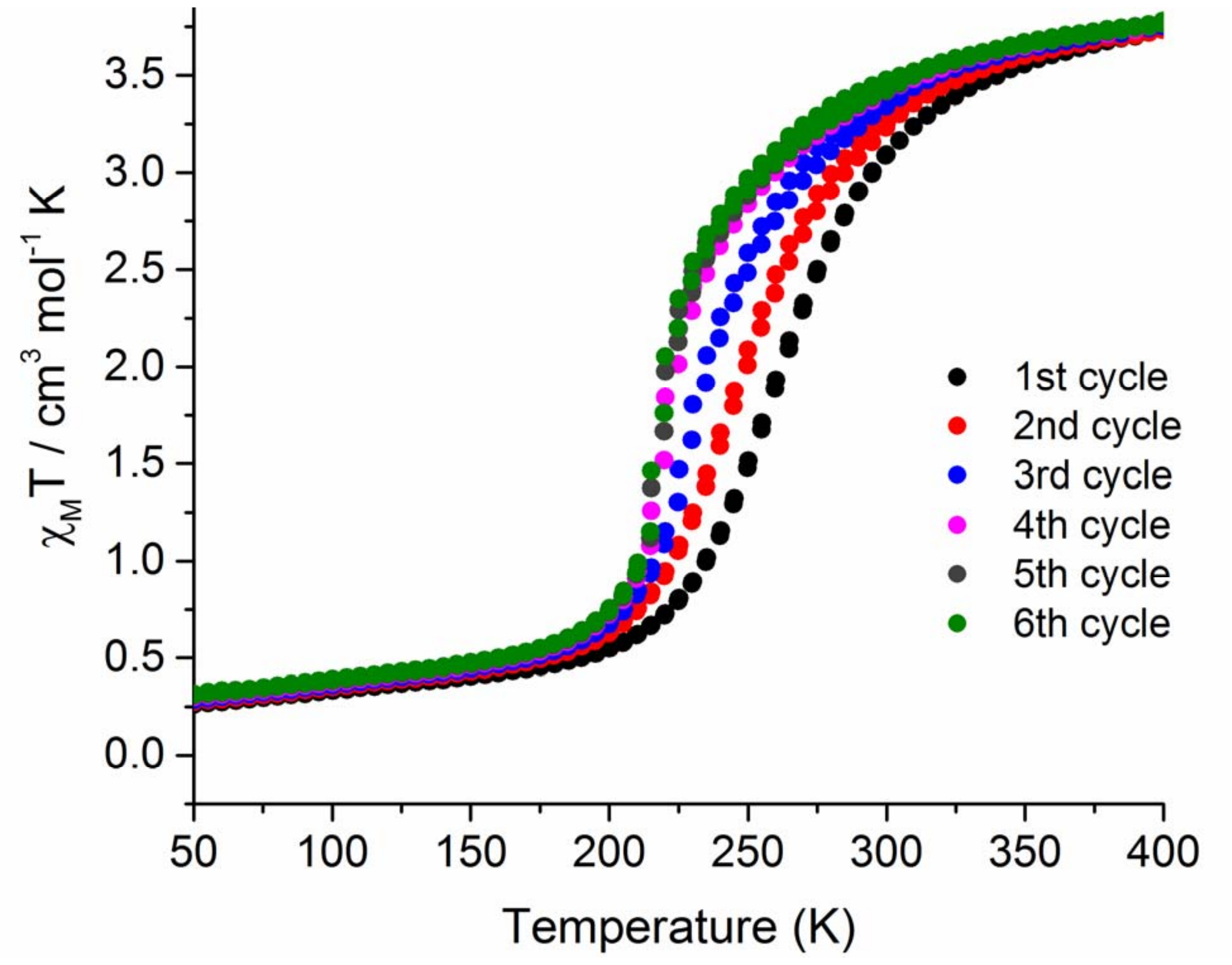

Figure S22. *$\chi_M T$ of **1**·n(THF) (where $0 \leq n \leq 1$) as a function of temperature for 300→50 K and then repeated cycles of 50↔400 K showing the solvent dependence of spin crossover in this compound. Inset: A zoom in over the spin transition seen for the 5th and 6th cycles, showing a slight hysteresis. Note: n is shown to be 1 for the first cycle and 0 for the 5th cycle by TGA. Data were collected in* ***settle mode.*** *MW(**1**·THF) used in all calculations.*

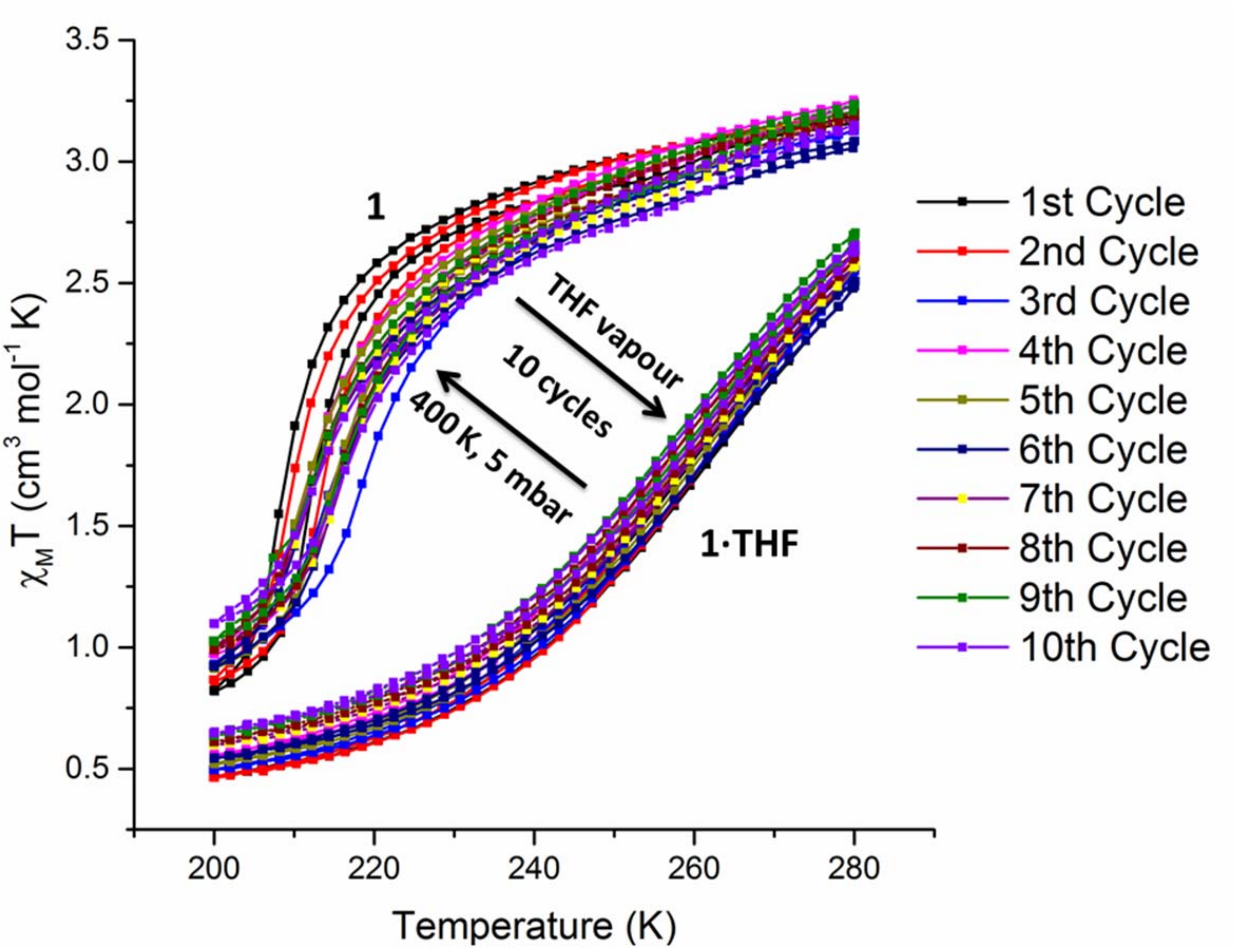

Figure S23. *$\chi_M T$ as a function of temperature from 280→200→280 K as **1**·THF is heated at 400 K for two hours to give **1** then exposed to THF vapour for 2 hours to regenerate **1**·THF over 10 consecutive cycles. To collect these data **1**·THF was loaded into a PPMS holder and $\chi_M T$ measured in the Versalab PPMS in the range 280→200→280 K with a scan rate of 1 K min^{-1}. The temperature was then ramped to 400 K at 5 K min^{-1} and held at that temperature for 2 hours. After this time the temperature was ramped to 280 K at 5 K min^{-1} and $\chi_M T$ measured in the Versalab PPMS in the range 280→200→280 K with a scan rate of 1 K min^{-1}. The sample was then transferred from the PPMS holder to a glass sample tube and subjected to THF vapours. The process was then repeated. Due to small sample losses between the PPMS holder and glass sample tube for each cycle we stoped after measuring 10 cycles. Before each Versalab PPMS measurement the sample was reweighed and this mass used for calculation of $\chi_M T$ for that cycle (the mass of the sample on the first cycle was 11.7 mg and it was 6.4 mg after the 10 cycles: we estimate an absolute weighing error of +/- 0.2 mg in each case). The molecular weight used in the data analysis was **1**·THF (870.8 g mol^{-1}) for samples after THF vapor exposure (as they are **1**·THF after that treatment) and **1** (792.7 g mol^{-1}) for samples after heating at 400 K (as they are solvent free after that treatment).*

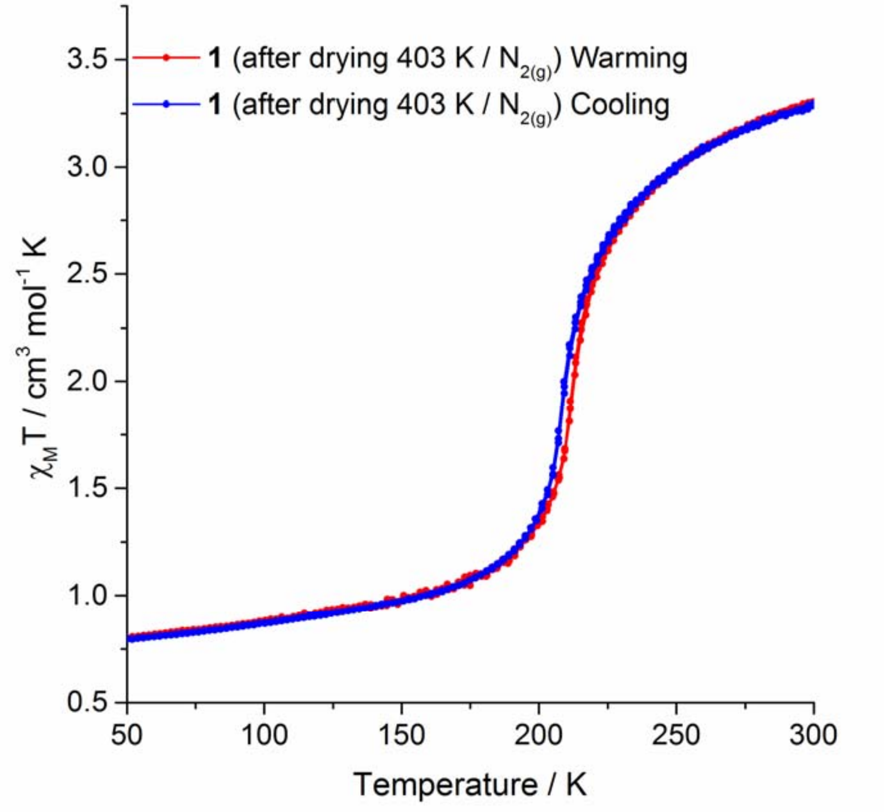

Figure S24. *Three cycles of $\chi_M T$ as a function of temperature on (dry)* ***1*** *show a consistent response. Data were collected in* ***settle mode.***

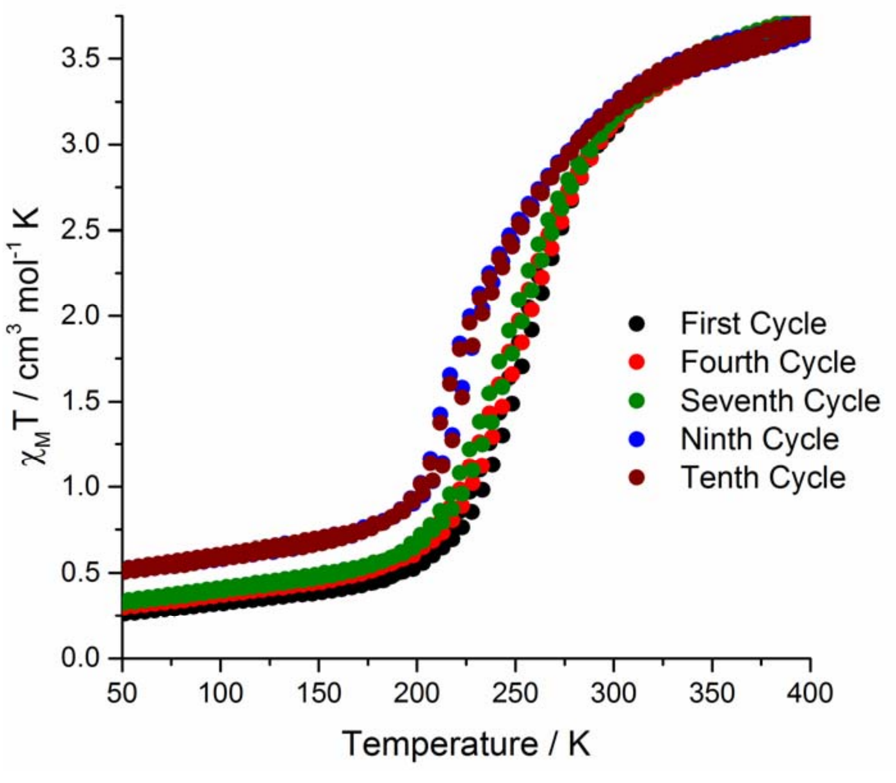

Figure S25. *Magnetic moment as a function of temperature for* ***1***$\cdot CHCl_3$ *as the sample is ramped to from 50↔400 K over multiple cycles in* ***settle mode****. The first cycles showed only very slight shifting of the* $T_{½}$ *so the temperature was held constant at 400 K for 1 hour before the ninth and tenth cycles. Data were collected in* ***settle mode****. MW(***1***$\cdot CHCl_3$) used in all calculations.*

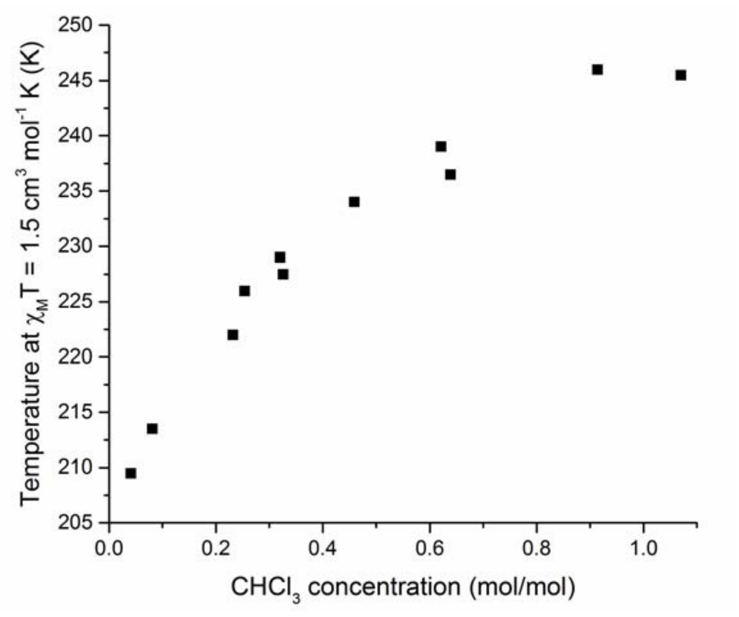

Figure S26. *A plot of mol fraction $CHCl_3$ as a function of the temperature at which $\chi_M T = 1.5$ cm^3 mol^{-1} K. The $\chi_M T$ data for this plot were collected in sweep mode with a scan rate of 1 K min^{-1} and the temperature shown is the average of that observed in the heating and cooling directions. See page S7 for a full description of the protocol used.*

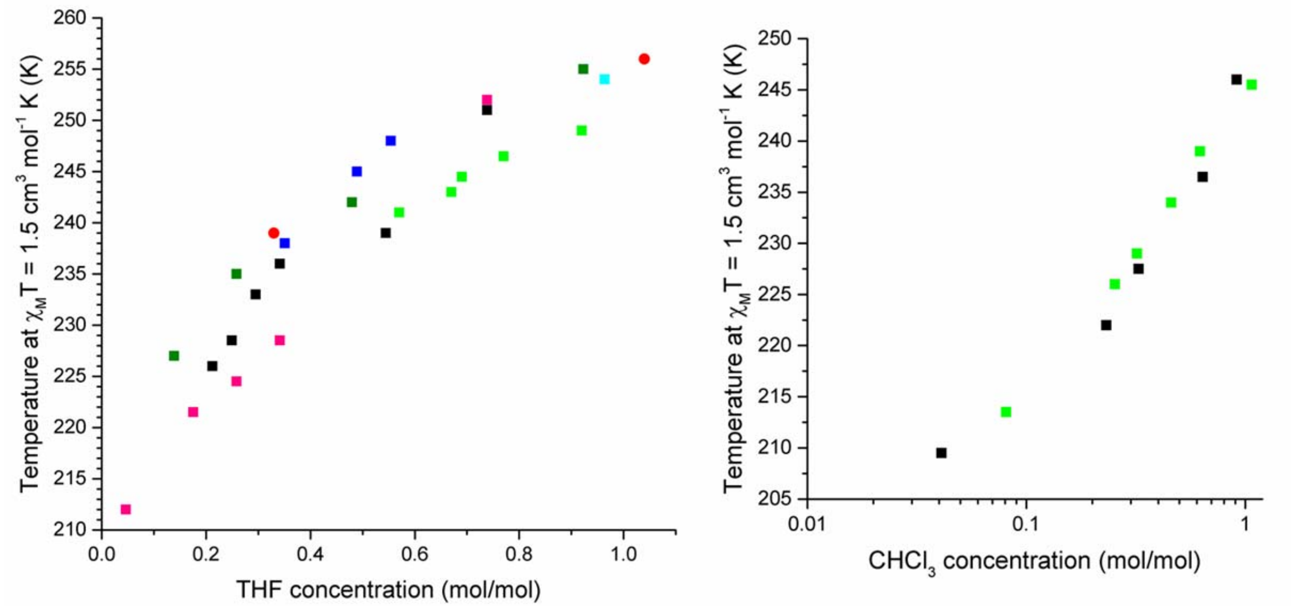

Figure S27. *Plots of mol fraction THF (left) and $CHCl_3$ (right) as a function of the temperature at which $\chi_M T = 1.5$ cm^3 mol^{-1} K (approximates the $T_{½}$). Colour of data points: Each colour corresponds to data for a particular batch of compound: several different batches of each compound were probed as detailed on page S7. The red circular data points represent a sample which was only heated once and then the entire sample used for TGA: this gave a result consistent with the samples in which only a small fraction of the original solvated sample was used due to previous removal of small TGA samples (blue and black squares), all three of them have a similar value of n and a $T_{½}$ of approximately 237 K, demonstrating that this method is not biased by inhomogeneity. Left: The three (red, dark green and cyan) data points of highest THF concentration (n approximately 1) represent 'as prepared, isolated and briefly dried', solvated, samples.Right: The two (black and green) data points of highest $CHCl_3$ concentration (n approximately 1) represent 'as prepared, isolated and briefly dried', solvated, samples.*

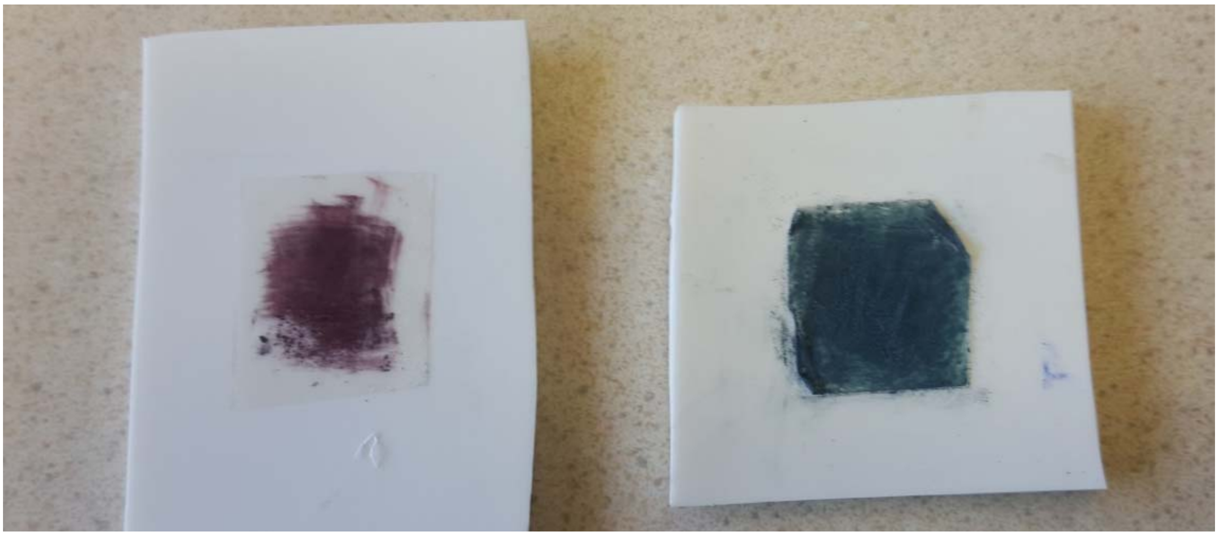

Figure S28. *Sample mounting method used for collection of the solid-state visible reflectance spectra. The sample on the left is* **1** *after drying at 403 K under a $N_{2(g)}$ flow. The sample on the right was initially treated in the same way, then exposed to THF vapour for 4 hours to regenerate* **1***·THF (shown) - this is the sample that was used to give the blue, pink and green lines in Figure S19.*

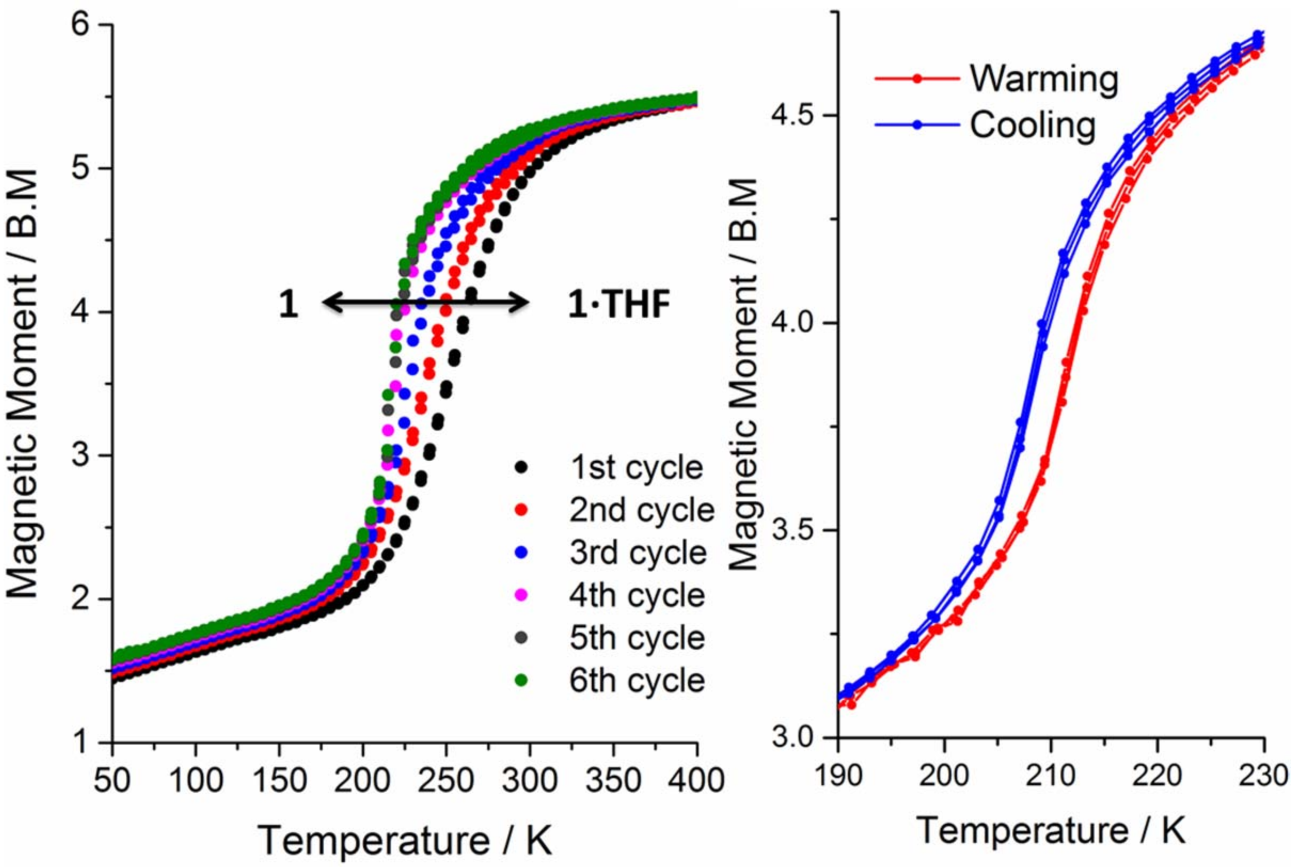

Figure S29*. The same data as shown in Figure 3, but with all calculations done using MW(***1***·THF).*

References

1. M. H. Klingele and S. Brooker, *Eur. J. Org. Chem.*, 2004, 3422-3434.
2. R. W. Hogue, R. M. Miller, N. G. White, H. L. C. Feltham, G. N. L. Jameson and S. Brooker, *Chem. Commun.*, 2014, **50**, 1435–1437.
3. O. Diffraction, *CryAlisPRO*, (2011).
4. G. M. Sheldrick, *SADABS. Empirical absorption correction program for area detector data*, (1996), Göttingen.
5. CrysAlisPro, *CrysAlisPro*, (CrysAlisPro) Agilent Technologies Yarnton, Oxfordshire.
6. G. M. Sheldrick, *Acta Crystallogr., Sect. A: Found. Crystallogr.*, 2008, **A64**, 112-122.
7. *X-Seed* (In press), J. Supramol. Chem.
